# HLA Class-II Associated HIV Polymorphisms Predict Escape from CD4+ T Cell Responses

**DOI:** 10.1371/journal.ppat.1005111

**Published:** 2015-08-24

**Authors:** Nathan Erdmann, Victor Y. Du, Jonathan Carlson, Malinda Schaefer, Alexander Jureka, Sarah Sterrett, Ling Yue, Dario Dilernia, Shabir Lakhi, Jianming Tang, John Sidney, Jill Gilmour, Susan Allen, Eric Hunter, Sonya Heath, Anju Bansal, Paul A. Goepfert

**Affiliations:** 1 Department of Medicine, University of Alabama at Birmingham, Birmingham, Alabama, United States of America; 2 Microsoft Research, Los Angeles, California, United States of America; 3 Department of Pathology and Laboratory Medicine, Emory University, Atlanta, Georgia, United States of America; 4 Zambia Emory Research Project, Lusaka, Zambia; 5 La Jolla Institute for Allergy & Immunology, La Jolla, California, United States of America; 6 International AIDS Vaccine Institute and Imperial College, London, United Kingdom; University of North Carolina at Chapel Hill, UNITED STATES

## Abstract

Antiretroviral therapy, antibody and CD8^+^ T cell-mediated responses targeting human immunodeficiency virus-1 (HIV-1) exert selection pressure on the virus necessitating escape; however, the ability of CD4^+^ T cells to exert selective pressure remains unclear. Using a computational approach on HIV *gag*/*pol*/*nef* sequences and HLA-II allelic data, we identified 29 HLA-II associated HIV sequence polymorphisms or adaptations (HLA-AP) in an African cohort of chronically HIV-infected individuals. Epitopes encompassing the predicted adaptation (AE) or its non-adapted (NAE) version were evaluated for immunogenicity. Using a CD8-depleted IFN-γ ELISpot assay, we determined that the magnitude of CD4^+^ T cell responses to the predicted epitopes in controllers was higher compared to non-controllers (p<0.0001). However, regardless of the group, the magnitude of responses to AE was lower as compared to NAE (p<0.0001). CD4^+^ T cell responses in patients with acute HIV infection (AHI) demonstrated poor immunogenicity towards AE as compared to NAE encoded by their transmitted founder virus. Longitudinal data in AHI off antiretroviral therapy demonstrated sequence changes that were biologically confirmed to represent CD4^+^ escape mutations. These data demonstrate an innovative application of HLA-associated polymorphisms to identify biologically relevant CD4^+^ epitopes and suggests CD4^+^ T cells are active participants in driving HIV evolution.

## Author Summary

In HIV, CD4^+^ T cells are best known as the primary targets of infection. Although emerging data has suggested a more active role in viral pathogenesis, the CD4^+^ T cell population remains relatively understudied. Using a novel computational approach, we predicted 29 different epitopes with mutations that potentially represent escape from CD4^+^ T cell responses. The predicted escaped epitopes were found to be less immunogenic than the wild type forms, suggesting that the identified escapes allow HIV to reduce its visibility to the immune system. Using longitudinal samples, we were able to show CD4^+^ T cells driving viral escape following acute infection. Overall, these findings significantly expand our knowledge of how CD4^+^ T cells can exert HIV control and influence HIV evolution, providing important implications to future vaccine development strategies.

## Introduction

The human immunodeficiency virus-1 (HIV-1) has the capacity to escape pressure exerted upon it by a number of factors including antiretroviral therapy (ART), antibody and CD8^+^ T cells [1–4]. Escape implies a level of viral suppression sufficient to provide selection pressure for mutant genomes to emerge that are no longer sensitive. This has been clearly demonstrated with ART where combination therapy has been shown effective in controlling HIV [5]. HIV-specific CD8^+^ T cell and neutralizing antibody responses drive HIV escape and both have been demonstrated to be important in controlling HIV infection [4,6–8]. Conversely, other innate and adaptive immune responses have either not been shown to drive HIV escape or have been only demonstrated to infrequently drive the process calling into question the utility of such responses in overall viral control [9]. CD4^+^ T cell responses have previously been associated with viral sequence changes, but the scope and relevance of CD4^+^ T cells on viral control has remained unclear [10–12]. Recently, viral escape in response to CD4^+^ T cell responses was definitively demonstrated in the SIV model, suggesting the potential for CD4^+^ T cells to mediate HIV control [13].

As CD4^+^ T cells are the primary targets of HIV infection, their contribution to the adaptive immune response targeting HIV-1 has been relatively understudied. While the role of these cells in providing help to B and CD8^+^ T cells is well documented, a growing body of literature also implicates HIV-specific CD4^+^ T cells in exacting immunological control of HIV via direct antiviral effects [14–16]. This newly attributed function for CD4^+^ T cells should, if of sufficient efficacy, select for mutations that allow the virus to evade immune recognition. Using a cohort of HIV-1 subtype C chronically-infected individuals for whom the HIV sequences (*gag*, *pol* and *nef*) and HLA-II allelic data were derived, we identified amino acid changes occurring at the population level that were disproportionately associated with specific HLA-II alleles. These HLA class II-associated HIV polymorphisms (HLA-AP) were used to predict epitopes encompassing them. Based on this adaptation nomenclature, 2 groups of epitopes were generated: **non-adapted epitopes** (NAE), epitopes without any evidence of HLA adaptation; and **adapted epitopes** (AE), those containing an amino acid associated with adaptation to a specific HLA-II allele. NAE were demonstrated to be more immunogenic and with enhanced cytotoxic capabilities in eliminating HIV-pulsed targets. Furthermore, we temporally associated HIV-specific CD4^+^ T cell responses with the emergence of viral escape following acute HIV infection. Our findings demonstrate that HLA-AP can predict CD4^+^ T cell epitopes that escape and that this viral escape is more common than previously appreciated, suggesting a role for CD4^+^ T cell responses in HIV control.

## Results

### HIV-1 adaptation based on HLA class-II-associated HIV polymorphisms

Although HLA-I associated HIV polymorphisms have been used extensively to identify signatures of CD8^+^ T cell induced escape [17–20], application of a similar methodology for HLA-II associations has not previously yielded any HIV-1 polymorphisms. Using sequences from HIV-1 *gag*, *pol*, and *nef*, and HLA-II allelic data from the Zambian cohort of 348 chronic HIV subtype C infected patients, HLA-II specific adaptations at the population level were identified computationally. The approach yielded 29 unique polymorphisms predicted to represent escape mutations in the context of a CD4^+^ T cell epitope (Table 1).

**Table 1:** List of HLA-II associated HIV-1 polymorphisms.

Protein	Position^a^	HLA-II polymorphism	Non-adapted^b^	Consensus^c^	HLA-II^d^
Gag	112	K	-	K	DQB1*06:03
Gag	147	L	I	I	DQB1*02
Gag	247	I	-	I	DQB1*06
Gag	339	S	P	P	DRB1*13
Pol	17	H	S	S	DQB1*02:01
Pol	68	T	S	T	DQB1*05
Pol	161	E	D	E	DQB1*04:02
Pol	208	D	E	E	DQB1*05
Pol	215	I	V	V	DRB1*08
Pol	333	V	I	I	DRB1*01:02
Pol	362	K	Q	Q	DQB1*03
Pol	430	K	-	K	DQB1*06:02
Pol	433	Y	V	V	DQB1*02:02
Pol	490	D	Q	Q	DRB1*10:01
Pol	499	D	E	E	DRB1*09:01
Pol	815	F	Y	F	DQB1*03:03
Pol	984	R	K	R	DQB1*06:04
Nef	16	I	V	V	DQB1*02:01
Nef	20	L	M	M	DQB1*03
Nef	24	A	E	E	DRB1*03
Nef	35	R	Q	R	DQB1*06:11
Nef	45	N	S	S	DQB1*02:01
Nef	88	G	S	S	DQB1*04
Nef	104	Q	K	Q	DQB1*05
Nef	135	F	Y	Y	DQB1*06:02
Nef	157	S/T	N	N	DRB1*11:01/DQB1*02:01
Nef	168	L	M	M	DQB1*04:02
Nef	188	G	R	R	DQB1*06:02
Nef	192	R	H	H	DQB1*02:01

^a^Amino-acid (AA) position based on HXB2 numbering

^b^The corresponding non-adapted AA at the polymorphic position; dash indicates lack of non-adapted AA predicted at that position

^c^The corresponding consensus HIV-1 clade B AA at the polymorphic position

^d^Predicted HLA-II allele associated with this AA polymorphism

### CD4^+^ T cell epitopes predicted by HLA-II associated HIV polymorphisms are immunogenic during chronic HIV infection

**Fig 1:**
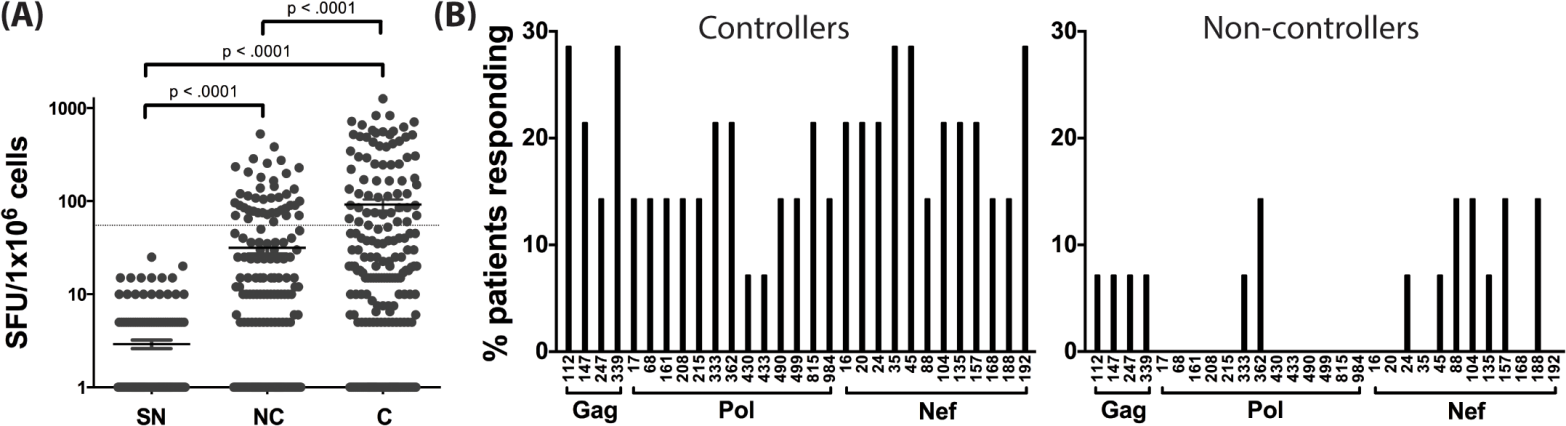
Novel HLA-II-restricted HIV-1 specific epitopes stimulate CD4^+^ T cell responses during chronic HIV infection. (A) Peptide pools containing the predicted CD4^+^ T cell epitopes (NAE and AE forms) were tested among HIV seronegative individuals (SN = 10), non-controllers (NC = 14), and controllers (C = 14) in an IFN-γ ELISpot assay. The magnitude of IFN-γ response (SFC/10^6^ PBMC) for all three groups is shown. Each dot represents a response to a single peptide pool, the dotted line demarcates the threshold for a positive ELISpot response (>50 SFC/10^6^). Mann-Whitney U test was used to calculate statistical significance, with error bars representing the SEM. (B) The percentage of patients responding to single peptide (NAE and AE forms) stimulations encompassing the 29 unique HLA-II associated polymorphic sites (shown by the numbering position within each protein, X-axis) as measured in an IFN-γ ELISpot assay is indicated for controllers (middle panel) and non-controllers (right panel).

Using the predicted class II polymorphisms, their respective HLA-II allele, and an *in silico* prediction algorithm [21], we generated a panel of peptides containing potential CD4^+^ T cell epitopes. Epitopes containing a predicted polymorphism represent an adapted epitope or AE, while their non-adapted (NAE) counterpart contains an amino acid with no evidence of adaptation, frequently the consensus sequence (S3 Table). Immunogenicity has not previously been defined for the predicted epitopes, and of the 29 unique prediction sites, 12 are entirely novel with no overlapping epitopes previously described (www.hiv.lanl.gov, HIV Molecular Immunology Database). For the remaining 17, partially overlapping epitopes, restricted by other HLA-II alleles, were reported. We tested the immunogenicity of these AE and NAE in a CD8-depleted IFN-γ ELISpot assay. Depletion of CD8^+^ cells prior to aliquoting cells was equally efficient when performed on PBMCs from both controller or non-controller donors. Thus, the number of CD4^+^ T cells placed in ELISpot wells was similar for all donors tested (S1 Fig). We first tested pools of peptides containing the identified AE and NAE to screen for responses in 10 HIV seronegative (SN) donors, 14 chronic HIV-infected (CHI) patients with viral loads >10,000 copies (Non-Controllers, NC), and 14 CHI patients with viral load <2,000 copies (Controllers, C) (Tables 2 and S1). Although there were differences in the HLA-II alleles represented in the controllers and non-controllers, this did not reach statistical significance. Initial screening demonstrated HIV-specific CD4^+^ T cell responses to these peptide pools in CHI patients. The magnitude of response by controllers was significantly greater as compared to both seronegative controls and non-controllers (Fig 1A). We observed each predicted CD4^+^ epitope to be immunogenic in at least one tested individual. Among controllers, we detected a response to each of the 29 tested epitopes; in contrast, we were only able to detect responses to 13 (45%) of the epitopes among all non-controllers (Fig 1B). A total of 8 patients (5/14 controllers and 3/14 non-controllers) had a confirmed mapped response to at least one tested epitope. For 43% of the mapped responses, the donor had the predicted HLA-II allele, whereas the remaining 57% of the time, the donor had a positive response despite not having the predicted allele.

**Table 2:** Summary of clinical features and demographic characteristics of the chronic cohort.

Group	HIV controller (C)	HIV non-controller (NC)
**Number of males**	10/14 (71%)	13/14 (93%)
**Race** ^**a**^		
CAU	3/14 (21%)	8/14 (57%)
AA	11/14 (79%)	6/14 (43%)
**Risk group** ^**b**^		
MSM	6/14 (43%)	12/14 (86%)
Heterosexual	6/14 (43%)	1/14 (7%)
IVDU	2/14 (14%)	1/14 (7%)
**Clinical** ^**c**^		
CD4*	853	465
Plasma VL**	194	25500

^a^CAU = Caucasian; AA = African-American

^b^MSM = Men who have sex with men; IVDU = IV drug user

^c^Absolute CD4 counts (cells/uL) and plasma VL (HIV-1 RNA copies/mL) are represented as median values; all patients are ART naïve.

*, p = 0.001 between controller and non-controller CD4 counts

**, p < 0.0001 between controller and non-controller plasma VL

To evaluate the accuracy of the predicted epitopes and their associated HLA-II alleles, we sought to confirm that at least some of the observed positive responses were presented to the CD4^+^ T cells by their computationally predicted HLA-II alleles. To do so, we used single HLA-II allele transfected RM3 cell lines as antigen presenting cells (APC) and *in vitro* expanded CD4^+^ T cell lines as effectors [22,23]. CD8-depleted PBMCs from two donors previously identified to have a positive *ex vivo* response were expanded and then exposed to peptide-pulsed APC expressing either the HLA-II allele associated with the adapted polymorphism or a mismatched allele. In these cases (S2 Fig), the predicted HLA-II restriction was shown to match the relevant allele of the APC, as the highest magnitude IFN-γ ELISpot response was detected when the peptide was presented by an RM3 cell line expressing the polymorphism-associated HLA-II allele.

### Adapted epitopes (AE) are poorly immunogenic in chronic infection

**Fig 2:**
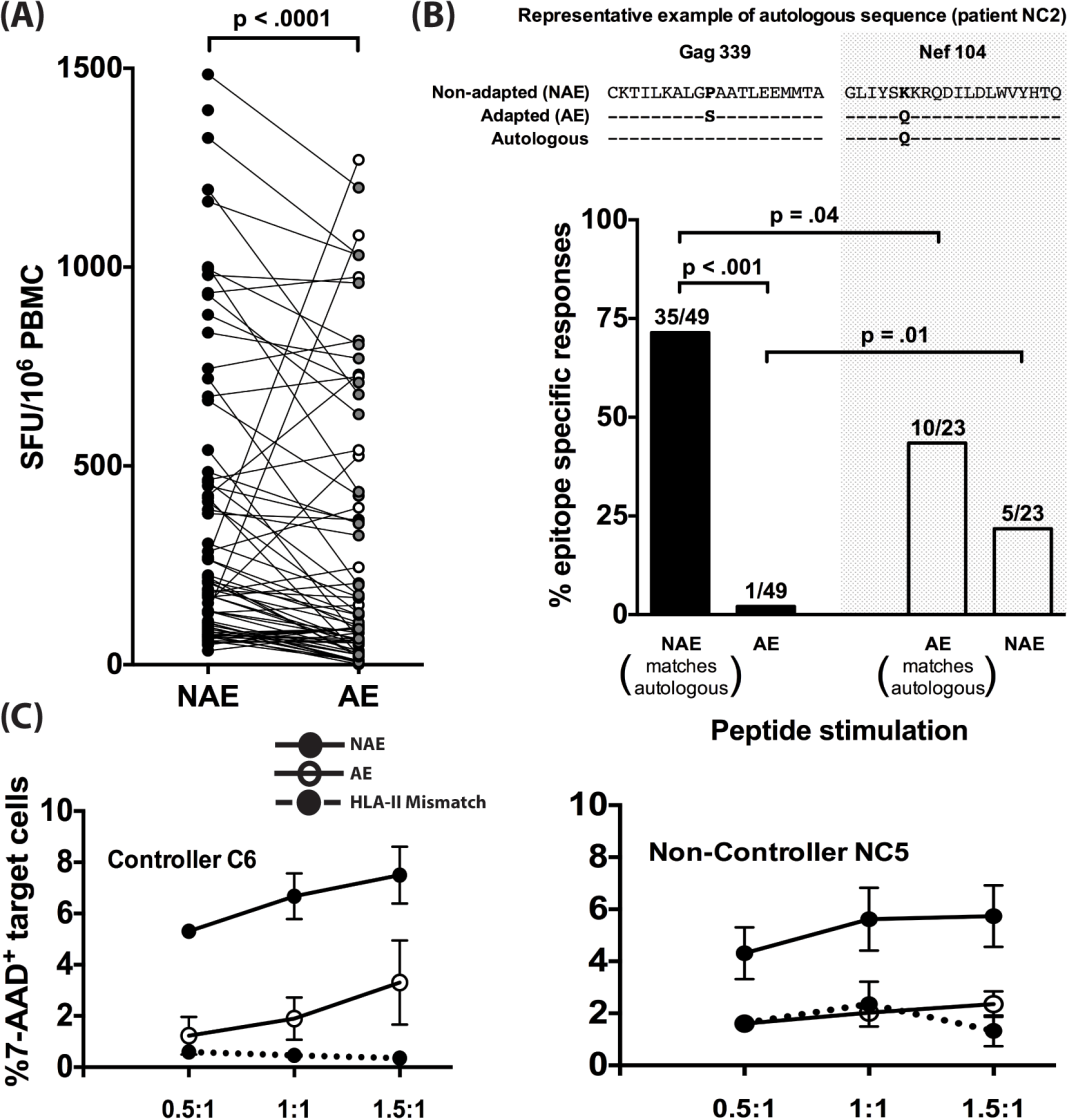
Adapted CD4^+^ T cell epitopes have reduced immunogenicity in chronic infection. (A) Magnitude of CD4^+^ T cell responses to NAE vs. AE evaluated in PBMC obtained from 8 (5 controllers and 3 non-controllers) HIV-1 chronically-infected donors using an IFN-γ ELISpot assay is shown. Each dot represents a single NAE or AE response. Filled and open dots indicate AE responses that are lower or higher in magnitude, respectively, as compared to their paired NAE responses. Wilcoxon matched-pairs signed rank test was used to determine statistical significance. (B) The dominant autologous viral sequences from 5 chronically infected patients (NC1, NC2, NC5, C4, and C6) were obtained. On an epitope basis, each sequence was then compared to see whether it matched an NAE or an AE. Examples of an NAE (Gag339) and AE (Nef104) matching the autologous sequence are shown. Polymorphic amino acids in viral sequences are bolded. Overall frequency of NAE and AE responses (N = 5) as determined in an IFN-γ ELISpot assay in donors with autologous sequence matching either NAE or AE is shown. The fraction on top of each bar indicates the number of positive responses out of the total number of peptides tested. (C) CD4^+^ T cells expanded 10 days *in vitro* with sequence-matched and mismatched peptides were used as effectors and CD4^+^ T cells from autologous and HLA-II mismatched individuals, pulsed with peptides of the same specificity as the effectors, were used as targets at various E:T ratios of 0.5:1, 1:1, and 1.5:1 for 24 hrs. Effector CD4^+^ T cell-mediated killing was determined by the percentage of 7-AAD^+^ targets in the presence of effector adjusted relative to % 7-AAD^+^ target cells without any effectors (E:T of 0:1). Graphical depiction comparing NAE and AE-specific CD4^+^ T cell induced killing is shown for a controller (C6) and a non-controller (NC5).

We next investigated differences in immunogenicity between adapted and non-adapted epitopes. Each identified polymorphism represents a prediction of HLA-II-mediated immune pressure at a single amino acid. Based on this, we hypothesized reduced immunogenicity for AE as compared to NAE. We compared the magnitude of positive IFN-γ responses from the ELISpot assays for NAE/AE pairs from CD8-depleted PBMC taken from 8 chronically-infected donors. Of the 70 pairs tested, 49 responses were found to have a reduced AE magnitude as compared to the observed NAE response (p<0.0001 by Wilcoxon ranked pairs) (Fig 2A).

The finding that the NAE induced a higher magnitude of responses compared to their AE counterparts can be explained by at least two different scenarios. First, as it is well known that autologous viral sequences are better at eliciting T-cell responses [24], the limited AE immunogenicity could reflect chronically-infected individuals having virus encoding a predominance of NAE. An alternative explanation is that AE are the result of host immune pressure on NAE epitopes and subsequent viral escape. To explore these alternatives, we performed population viral sequencing, determining the dominant form of the virus in the 5 CHI individuals who demonstrated responses to the predicted epitopes and for whom plasma/PBMC samples were available (2 controllers and 3 non-controllers, S4 Table). We determined whether the dominant autologous viral sequence matched the NAE or AE at the amino acid from the identified polymorphic site (Fig 2B). The highest frequency of responses (71%) was observed in patients whose autologous sequence matched the non-adapted form and who were subsequently stimulated with NAE (p = 0.04 compared to AE). Conversely, in 5/23 cases (22%) where the dominant viral quasispecies encoded the AE form, NAE was found to be immunogenic, which was not significantly different from the immunogenicity of the matching AE (10/23 or 43%).

Prior work has shown CD8^+^ T-cell mediated antigen sensitivity (functional avidity) is a reliable predictor of viral control [25,26]. To determine whether this was also evident in epitope-specific CD4^+^ T cells, we determined the antigen sensitivity of immunogenic NAE and AE where the epitope sequence matched the dominant viral species present in the respective patients. We tested CD8-depleted PBMCs from five different patients with 6 NAE/AE responding pairs of peptides. The functional avidity did not significantly differ between NAE and AE, with a higher NAE magnitude observed only at the lowest 10^−7^ M peptide concentration tested (S3 Fig).

Since polyfunctional CD4^+^ T cells, especially those making granzyme A, have been implicated in viral control [27], we next evaluated the functionality of antigen-specific CD4^+^ T cells using multiparametric flow. PBMCs from 4 CHI patients were stimulated *ex vivo* and evaluated in an intracellular cytokine staining assay. We observed similar up regulation of cytokines and cytotoxic factors by CD4^+^ T cells for both NAE and AE specific responses (S4 Fig).

We next wanted to assess whether both NAE and AE-specific CD4^+^ T cells can effectively kill HIV targets. Autologous as well as HLA-II mismatched CD4^+^ T cell targets (as a negative control) were pulsed with either NAE or AE peptide and then co-cultured with the respective NAE or AE effector cells. Using a 7-AAD assay, we observed higher CD4-mediated killing of autologous NAE-pulsed targets, relative to HLA-II mismatched targets, but killing of AE-pulsed targets was significantly impaired (Fig 2C).

### CD4^+^ adapted epitopes (AE) are poorly immunogenic during acute infection

**Fig 3:**
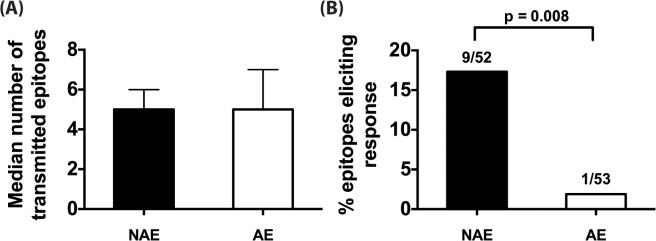
CD4^+^ T cell adapted epitopes (AE) are poorly immunogenic during acute infection. (A) Median number of NAE and AE encoded by transmitted founder virus (TFV) that infected each of the 11 acute patients is indicated. Error bars represent the interquartile range of the median. (B) For each unique HLA-II predicted epitope, relevant NAE or AE peptide matching the determined TFV sequence was tested in IFN-γ ELISpot. Percentage of NAE or AE that was immunogenic in at least one patient is shown. Fractions represent number of epitopes that elicited a response / total number of epitopes tested. Fisher’s exact test was used to determine significance.

A limitation to studies in chronically-infected HIV patients is that they harbor highly heterogeneous HIV-1 variants. Therefore, it is not possible to ascertain whether an immune response reflects a *de novo* response elicited by that epitope or cross presentation of an epitope variant. To accurately identify epitope-induced responses, we used single genome amplification technique [28–30] to obtain transmitted founder virus (TFV) sequences from the plasma of 11 clade B acutely infected patients (Fiebig stages I-III, S2 Table). Taking into consideration each donor’s HLA-II alleles, we determined the number of NAE and AE encoded by TFV that established infection in each acute patient (number of encoded NAE and AE for a representative patient is shown in S5 Table). While the number of transmitted NAE and AE varied from individual to individual (range of 2–10 for NAE, 2–7 for AE), the median numbers encoded by the TFV per infected individual were similar (Fig 3A), indicating that transmission of CD4^+^ AE variants is relatively common. Overall, in these 11 patients, we identified 52 and 53 predicted NAE and AE, respectively, encoded in the TFV. After stimulating each patient’s PBMC with the appropriate TFV-encoded epitopes (NAE or AE) in an IFN-γ ELISPOT assay, only 1/53 AE peptide elicited an immune response versus 9/52 NAE specific responses (p = 0.008) (Fig 3B). Each predicted polymorphism represents a possible CD4^+^ T-cell escape mutation, as AE are poorly immunogenic even when encoded by the TFV. The presence of AE in the TFV, therefore, possibly represents CD4^+^ T cell epitopes that have escaped in a prior host.

### CD4^+^ T cell responses are temporally associated with predicted escape following acute infection

**Fig 4:**
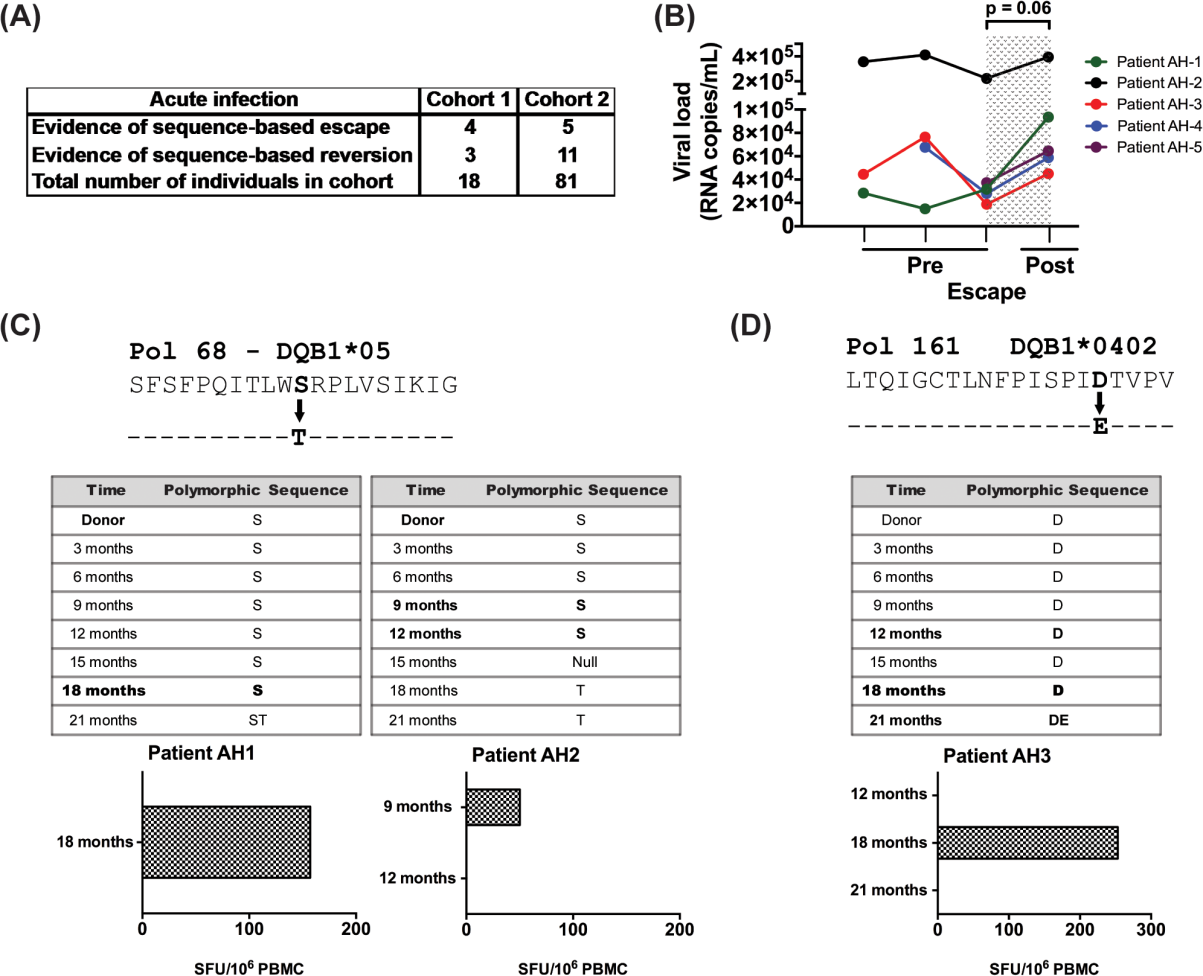
Evidence for CD4 T-cell escape following acute infection in HIV-1 clade C infected patients. (A) HIV sequence data and HLA alleles were obtained from two distinct cohorts of acutely infected individuals followed longitudinally (N = 99) and were used to determine the frequency of escape and reversion. (B) Longitudinal changes in viral load are shown at the CD4^+^ T cell pre-escape and escape time points for 5 donors on whom data was available. Patient AH-5 is from cohort 1 and the remaining 4 are from cohort 2. Statistical significance was determined using Wilcoxon matched-pairs signed rank test. (C) Using an IFN-γ ELISpot assay, responses to the NAE and AE forms of Pol 68 epitope were evaluated using PBMCs from donors AH-1 and AH-2. (D) PBMCs from patient AH-3 were expanded 10 days in culture and then stimulated with NAE and AE forms of Pol 161 epitope in IFN-γ ELISpot assay. Bold indicates time points when PBMCs were available for testing.

We next analyzed longitudinal sequence data from two distinct acute infection cohorts to look for evidence of longitudinal HIV sequence changes based on HLA-II associated HIV-1 polymorphisms. As shown in Fig 4A, we observed 9 changes reflective of escape and 14 of reversion among the 99 patients in these cohorts. In each of the 5 patients, where longitudinal viral load data (pre and post escape) were available, there was a non-significant increase in viral load at the time point immediately following escape (mean VL increase of 72,675 copies, SEM = 36,337, p = 0.06; Fig 4B), as compared to the time point preceding escape. Further analysis revealed no evidence of viral sequence changes at known CD8 polymorphic sites at these time points for each of the 5 donors reported and their respective repertoire of HLA-I alleles.

We next looked for biologic evidence of viral escape. Limited access to samples and diminished CD4^+^ T cell responses in cryopreserved specimens restricted these studies [31]; however, we identified samples from time points with evidence of viral escape at predicted polymorphic sites and tested CD8-depleted PBMCs for immunogenicity to the NAE and AE in 6 patients. We found biologic evidence of viral escape in 3 of these 6 donors, with 2 instances at the Pol 68 epitope (Fig 4C) and one at the Pol 161 epitope (Fig 4D). Based upon TFV sequence data, each individual was determined to have been infected with the NAE form at the polymorphic site before the AE became the dominant sequence at a later time point. In each case, NAE stimulation elicited a response from CD8-depleted PBMCs prior to the predicted escape. In AH-1, a robust ELISpot response to the NAE was observed immediately prior to viral escape at the 18-month time point; no response to the AE was observed. For this patient, samples from later time points were not available. In AH-2, a modest ELISpot response to the NAE was observed immediately prior to escape at the 9-month time point. At the subsequent 12-month collection time, the NAE response was lost; a response to AE at either time point was not observed. In AH-3 (Fig 4D), initial ELISpot screening was negative for the NAE and AE at time points 12, 18 and 21 months, however cultured ELISpot revealed evidence of the NAE but not AE response prior to viral escape at 18 months. This response was lost at the 21-month time point.

## Discussion

HIV-specific CD4^+^ T cells have been relatively understudied compared to other arms of the adapted immune response. Prior studies of HIV pathogenesis identified an association between preserved CD4^+^ T cell function and improved clinical progression [32], but a lingering question has been whether the observed CD4^+^ T cell function simply reflects immune preservation or that this cell population influences viral immune control. Recent studies have demonstrated HIV-specific CD4^+^ T cell responses correlate with viral control [27] as has been demonstrated with CD8^+^ T cells in the past [33,34]. Furthermore, emerging data indicate that HIV-specific CD4^+^ T cells with cytolytic potential are especially important in early viral control [35]. These works suggest that CD4^+^ T cells should force HIV escape mutations similar to what has been extensively observed for both CD8^+^ T cells and neutralizing antibodies. However, prior studies have suggested that CD4^+^ T cells exert only limited selective pressure on HIV [12,36–38]. Definitive CD4^+^ T cell escape has been reported in SIV, but this was only described in a single rhesus macaque at a single epitope [13].

Here we demonstrate multiple lines of evidence for HIV escape from CD4^+^ T cell responses. Similar to HLA-I associated HIV polymorphisms, HLA-II associations were used to predict 29 sites representing viral evolution. While we did not formally demonstrate that each of these predictions represented an escape, it is interesting that every one of the predicted epitopes was found to be immunogenic. Furthermore, non-escaped epitopes or NAE were more immunogenic than AE in chronic infection, a finding not explained by preferential infection by viruses encoding NAE. Moreover, AE were poorly immunogenic in acute HIV infection where limited viral diversity of the TFV ensures the identity of the epitope eliciting the observed immune response as either NAE or AE. Finally, in 2 cohorts of acutely infected patients followed off ART for 2 years, we see evidence for CD4^+^ escape and temporally associate three of these responses with subsequent escape. Interestingly, several of the polymorphisms identified in this study reverted following infection into hosts no longer able to target the epitope, suggesting CD4^+^ T cell-induced escapes impose a viral fitness cost as has been demonstrated extensively for CD8^+^ T cells. This observation is consistent with HLA-I-restricted escape in which protective alleles drive escape associations at more sites and with larger effect sizes, suggesting a model in which the presence of detectable selection pressure corresponds to effective CD8 or CD4-mediated killing [18]. In sum our data provide strong evidence for CD4^+^ T cell responses influencing viral evolution.

Despite the fact that HIV escapes from CD8^+^ T cells, neutralizing antibodies, and even NK cells [1–4,6,39], it is interesting that escape from CD4^+^ T cells has been much more difficult to document [10–12]. This may be partially attributable to the prominence of CD8^+^ T cell escape, thus making it difficult to separate the effect of this immune response from those induced by CD4^+^ T cells. Our findings suggest CD4-mediated escape happens on a larger scale than previously appreciated, although it is unlikely to be as prevalent as CD8-mediated escape. Another difficulty in defining HLA-II polymorphisms is that HLA class II alleles bind epitopes much more promiscuously compared to HLA-I, making it more difficult to assign the polymorphism to a specific HLA-II allele. In patients with chronic infection, we mapped positive responses in donors that had the model predicted HLA-II allele, but also observed responses in donors that did not have the predicted allele. In light of the promiscuity of HLA-II binding, it is not surprising that HLA-II alleles other than those predicted by the model presented here can also present the epitopes of interest. The promiscuous response of HLA-II alleles is almost certainly influencing HIV at polymorphic sites other than those predicted by this particular model, but these changes are obfuscated by the contribution of multiple alleles. Nevertheless, the observation of compromised immunogenicity of AE in the setting of acute infection was restricted to studies of donors expressing the predicted HLA-II alleles, thereby strengthening the likelihood that HLA-II associated HIV polymorphisms predict escape from CD4 T cells. The predictions were based on HIV (*gag*, *pol*, and *nef*) and HLA-II sequences in 348 Zambians with chronic HIV infection, and it seems likely that further polymorphisms can be identified by studying more individuals and additional HIV proteins [18–20,40,41].

Our findings convincingly demonstrate both that CD4^+^ T cells respond to the predicted panel of polymorphic epitopes, and viral sequence change to the adapted form compromises this response. Nevertheless, CD8^+^ T cell responses are highly prevalent in HIV infection, so to address the possibility that CD8 responses may be driving viral escape we looked for CD8^+^ T cell responses to peptides yielding positive CD4^+^ T cell responses in a flow-based assay. In 7 unique donors we tested 19 peptides and observed CD4^+^ but not CD8^+^ T cell responses to the peptides. We also noted that none of the HLA-II alleles for which we observed polymorphisms were in significant linkage disequilibrium with HLA-I alleles. The one exception was that DQB1*06 is in negative linkage with HLA-B*57, yet both are associated with a p24 polymorphism at position 247 (S6 Table). Indeed, the one individual who had a response to the predicted CD4 epitope was DQB1*06 positive and HLA-B*57 negative. These findings speak to the possibility that multiple immune parameters (in this case both CD4^+^ and CD8^+^ T cells) are driving viral evolution independently and further solidify our results.

Although the HLA-AP presented here were derived from a clade C infected African cohort, their immunogenicity was evaluated in both clade B and C infected samples. The consistent immunogenicity across clades suggests the predicted CD4 epitopes are shared to some extent in HIV-1 infection by different subtypes. Similar to HLA-I associated escape, site-specific viral escape may be maintained across clades [42]. Genome-wide association study (GWAS) has previously failed to identify HLA-II alleles that are associated with viral control, which may reflect the promiscuity of antigen presentation. Furthermore, unlike HLA-I alleles that encode a single heavy chain responsible for peptide loading and interaction with TCR, HLA-II must function as a heterodimer consisting of an alpha and beta chains. Association analyses facilitated by GWAS only assess one polymorphism at a time without testing their combinations; the ability of such an approach to accurately capture HLA allelic function is much less accurate for HLA-II alleles [43]. Prior work implicated gag-specific CD4^+^ responses as most relevant to viral control [27,44], whereas our predictions had limited associations within Gag. A prior investigation did attempt to identify HLA-II polymorphisms using a cohort approach [38]. This analysis was done in Gag-Protease and used a similar but distinct computational method [42]. By applying a revised method [18] and extending our analysis to the rest of Pol and Nef, we identified a number of polymorphisms that were then tested biologically for validity. The majority of our predictions were outside of Gag, which would not have been picked up in this prior analysis.

An important question arising from these studies concerns whether HIV-specific CD4^+^ T cells are playing a role in viral control. The fact that they are pressuring the virus to mutate to avoid CD4 responses certainly suggests that they do contribute to this process, albeit at a magnitude that is likely much less than CD8 responses. Furthermore, our findings demonstrate the ability of HIV-specific CD4^+^ T cells to induce viral escape and suggest that such escapes are not rare events. These results should serve as a springboard for future research to definitively determine the role of HIV-specific CD4^+^ T cells in viral control.

## Materials and Methods

### Ethics statement

Chronic and acutely-infected individuals from Zambia provided written, informed consent to the Zambia University Teaching Hospital ethics committee and the Emory University institutional review board (IRB) prior to collection of information. Additional patients were recruited from the University of Alabama at Birmingham Adult AIDS 1917 clinic after obtaining written, informed consent and approval from the IRB at UAB.

### Study cohorts

A cohort of 348 Zambian individuals chronically infected with HIV-1 subtype C had the *gag*, *pol*, and *nef* gene viral sequences determined by population sequencing. Virus from an additional 81 acutely infected individuals from the Zambian cohort was sequenced longitudinally and PBMC from five of these HIV-1 subtype C-acutely infected ART naïve Zambian patients were used in this study. These patients were recruited at the Zambia-Emory HIV Research Project (ZEHRP) in Lusaka, Zambia into the ZEHRP heterosexual transmission discordant couple cohort and the International AIDS Vaccine Initiative (IAVI) Protocol C; informed consent was obtained from all patients. HIV sequences were also obtained from 18 subtype-C acutely infected patients from the CHAVI cohort [29]. For the IAVI cohort, the earliest viral sequences were obtained approximately 1–2 weeks post-infection. For the Zambian cohort, the first sequences were obtained approximately 3 months post infection. Both cohorts are heterogeneous with regards to when escape or reversion occurred, as that is different for each individual. Escape was noted to occur as early as two weeks or as late as 24 months post infection; reversion occurred as early as 6 months or as late as 24 months post infection.

Cryopreserved PBMC samples from ART naïve HIV-1 clade B chronically infected patients (N = 28) (S1 Table) and acutely infected patients (N = 11) (S2 Table) were used in this study. The chronic cohort included HIV controllers (n = 14, VL <2,000 copies/mL) and HIV non-controllers (n = 14, VL >10,000 copies/mL); both off ART. Details on the demographics and clinical features of these chronically infected individuals are shown in Tables 2 and S1. Acute HIV infection was stratified by the Fiebig staging. Healthy HIV seronegative donors (N = 10) from the Alabama Vaccine Research Clinic were used as controls.

### HLA-II genotyping

High-resolution (4-digit) genotyping data for the Zambian cohort came from earlier work [43]. For additional genotyping, we applied two molecular techniques to define *HLA-DRB1* and *HLA-DQB1* alleles in local patients. The procedures for sequencing-based typing (Abbott Molecular Inc., Des Plaines, IL) and automated DNA hybridization with oligonucleotide probes (Innogenetics Inc., Alpharetta, GA) have been described elsewhere [45,46]. Assignment of 2-locus haplotypes was based on known patterns of linkage disequilibrium in African American and European American populations.

### HLA-II associated HIV-1 polymorphisms prediction

We identified HIV polymorphisms in HIV *gag*, *pol*, and *nef* that were statistically enriched in 348 chronically clade C infected, antiretroviral-naïve Zambian individuals expressing specific HLA-II alleles. We then performed phylogenetically corrected logistic regression as implemented in the PhyloD software [42]. This approach takes as input a protein-specific phylogenetic tree (as inferred by Phyml 3.0 [47]), HIV sequences, and putative predictor variables. Separate phylogenetically corrected logistic regression models are inferred for each amino acid at each site, using forward selection to select features and computing p-values using likelihood ratio tests. Overall significance is reported as q-values, which estimate the false discovery rate associated with each p-value [48]; q<0.2 was chosen as a threshold for experimental follow up. Only features (amino acids at a given site, class I and class II alleles, at 2- and 4-digit resolution) that were observed in at least 3 (and at most N = 3) individuals were considered.

Our initial application of PhyloD closely followed previously published applications [18], with the exception that both class I and class II alleles were included as predictor variables. This approach yielded seven sites for which at least one amino acid was positively or negative associated with at least one HLA-II allele, called a “class-II-associated site”. An important caveat to this approach is that amino acid mixtures present a problem to logistic regression. Typically, PhyloD treats mixtures as uncertain observations by creating equally weighted fractional observations. However, HIV mixtures may represent positive evidence for incomplete selection. We therefore ran a second analysis in which all mixture amino acids were treated as positive observations of polymorphic amino acids. This analysis resulted in 16 class-II-associated sites. Although some of these sites overlapped with the initial eight sites, the specific alleles and amino acids differed, perhaps indicating different intensities of selection pressure. Finally, we reasoned that class I alleles may serve as the dominant source of selection pressure for most sites. We therefore reran the analysis (treating mixtures as noisy observations, as in the first analysis) such that forward selection preferentially added class I alleles and co-varying amino acids. Specifically, class I alleles and co-varying amino acids were greedily added to the model using a threshold of p<0.05. Class II alleles were considered only when no other amino acid or class I allele significantly improved model fit. This analysis resulted in nine class-II-associated sites, again with little overlap with the previous two analyses. Our overall analysis yielded 29 unique sites.

Because our aim in these statistical analyses was to guide experiments, we chose to follow up with all 29 class-II-associated sites that resulted from these three approaches. Using the consensus clade-B sequence, a 31 amino acid sequence was selected (15 amino acids upstream/downstream of the polymorphism). This sequence was then input into the Net MHCII prediction software (http://www.cbs.dtu.dk/services/NetMHCIIpan). Using the class-II allele associated with each unique polymorphism, a 20-mer amino acid sequence with the highest HLA-II binding affinity (IC50 < 500 nM) was selected for synthesis and immunogenicity testing. These polymorphism containing epitopes are henceforth referred to as adapted epitopes or AE. For each predicted adapted epitope, a corresponding consensus peptide (non-adapted epitope or NAE) was also synthesized. In those cases where the predicted adaptation mirrored the consensus, the next most common form of clade-B amino acid residue occurring at the polymorphic site was defined as the non-adapted form. This resulted in a panel of 70 total peptides that were used to assess CD4^+^ T-cell reactivity. For the initial response screening, the peptides were grouped into 17 pools with 4–5 peptides per pool.

### Peptide synthesis

Peptides encompassing the predicted CD4^+^ T cell epitopes and the identified amino acid polymorphisms were synthesized by New England Peptide (NEP) in a 96-array format and were reconstituted at 40mM in dimethyl sulfoxide and stored at -70°C until use.

### IFN-γ ELISPOT assay

A modified ELISPOT assay was performed as previously described [23,27]. Briefly, nitrocellulose plates were coated with anti-IFN-γ antibody overnight. The next day, PBMC were CD8-depleted using magnetic beads (Dynabeads CD8, Invitrogen), and the enriched CD4^+^ T cells were plated at 100,000 cells/well and were stimulated with the appropriate peptide pool or single peptide at a final concentration of 10uM for 40h at 37°C and 5% CO_2_. The cells were washed, and biotinylated anti-IFN-γ antibody was added to the plate for 2 hrs. After this incubation, streptavidin-alkaline phosphatase was added for 1hr before applying BCIP/NBT substrate for spot detection. The number of IFN-γ responses were enumerated using an ELISPOT plate reader (CTL S6 Ultra-V Analyzer) and the data normalized to Spot Forming Cells per million (SFC/10^6^). A positive response was defined as 50 SFC/10^6^ PBMCs or greater and at least 2.5 times background (unstimulated media only wells). Phytohemagglutinin (PHA) was used as a positive control. In certain cases where no detectable *ex vivo* response was apparent, CD8-depleted PBMCs were cultured in the presence of peptide (10uM), IL-7 (25ng/ml) and IL-2 (50IU/ml) for 7–10 days. Expanded antigen specific cells were then re-stimulated with peptide in an IFN-γ ELISpot assay as described above.

### Antigen sensitivity or functional avidity

To determine avidity, four 10-fold serial dilutions of peptides were used in an IFN-γ ELISPOT assay as described above. Antigen sensitivity was determined by the peptide concentration that elicited 50% of maximal IFN-γ response (EC_50_) for any given epitope.

### Intracellular cytokine staining (ICS)

Flow cytometry based ICS assay was done as previously described [35,49,50]. In brief, 10^6^ PBMCs were pulsed with peptide at 10uM in the presence of co-stimulatory antibodies (anti-CD28 and anti-CD49D) and anti-CD107a-FITC (all from BD Biosciences) for 2 hrs at 37°C. Monensin and brefeldin A (both from BD Biosciences) were then added and the cultures incubated for an additional 12 hrs. Next day, the cells were labeled with LIVE/DEAD cell dye (Invitrogen) and surface stained with anti-CD3-Pac Blue, anti-CD8-V500, and anti-CD4-Alexa 780 (both from BD Biosciences). The cells were permeabilized and labeled with anti-IFN-γ-Alexa 700, anti-IL-2-APC, anti-TNFα-PECy7, and anti-Granzyme A-PE (all from BD Biosciences). CD3 events greater than 100,000 were acquired on an LSR II (BD Immunocytometry Systems), and data were analyzed using FlowJo (version 9.6.4; TreeStar). Polyfunctionality analysis was performed using Boolean gating and SPICE and Pestle software (version 5.1; NIAID).

### HLA-II restriction using an IFN-γ based ELISPOT assay

HLA-II restriction assay was done essentially as described before [22,23]. In-vitro expanded CD4^+^ T cell lines were used as effectors and peptide pulsed RM3 cell line transfectants were used as antigen presenting cells (APC). In brief, CD8-depleted CD4^+^ T cells were cultured in the presence of IL-7 (5ng/ml), nevirapine (1ug/ml), IL-2 (50IU/mL) and peptide (5uM) for 14 days. The RM3 cell lines were propagated, pulsed with peptide (10uM) for 3 hrs and then washed thoroughly to get rid of excess peptide. The effectors (expanded CD4^+^ T cells) and the targets (peptide pulsed RM3 cell lines) were plated at 50,000 and 100,000 cells/well, respectively (i.e. 1:2 E/T ratio in an ELISPOT assay). The culture was then incubated for 24 hrs at 37°C and 5% CO_2_. We used HLA-II expressing RM3 cell lines relevant to the patient’s HLA-II allele and determined the IFN-γ production when these lines were either not pulsed or pulsed with a peptide that a) elicited a response previously and b) was predicted to be restricted by the same HLA-II as the HLA-II transfected cell line that was used as APC. We used two negative controls: an HLA-II mismatched irrelevant peptide and a cell line that did not express any of the patient’s HLA-II alleles.

### Viral RNA/genomic DNA extraction and cDNA synthesis

Viral RNA and genomic DNA were extracted from plasma (3 CHI donors) or PBMC (2 CHI donors) by using the QIAamp RNA and DNA mini kits (Qiagen, Valencia, CA), and cDNA synthesis was carried out using Superscript III (Invitrogen). Reverse transcription of extracted RNA was performed, in two stages: in the 1^st^ stage incubation was set at 50°C with 5U of reverse transcriptase (RT) for 1 hour in the presence of 0.5 mM of each dNTP, 5 mM DTT, 2U/μl RNaseOUT (RNase inhibitor), and 0.25 mM antisense primer; the 2^nd^ stage involved incubation at 55°C with an additional 5U of RT for 2 hours. Synthesis was initiated by reverse primer: 5’-ACTACTTAGAGCACTCAAGGCAAGCTTTATTG-3’ [51] and terminated by incubation at 70°C for 15 min, followed by 20 min at 37°C with 1μl RNase H. The cDNA was used immediately for near full-length HIV-1 PCR amplification.

Full-length HIV-1 genome amplification used forward primer 1.U5Cc—HXB2 positions 538–571–5’-CCTTGAGTGCTCTAAGTAGTGTGTGCCCGTCTGT-3’, and reverse primer 1.3’3’PlCb at HXB2 positions 9611–9642–5’-ACTACTTAGAGCACTCAAGGCAAGCTTTATTG-3’; the 2^nd^ round primers were: Forward primer 2.U5Cd at HXB2 positions 552–581–5’-AGTAGTGTGTGCCCGTCTGTTGTGTGACTC-3’ and reverse primer 2.3’3’plCb at HXB2 positions 9604–9636–5’-TAGAGCACTCAAGGCAAGCTTTATTGAGG-3’ [51]. PCR reactions were carried out by using the Q5 High-Fidelity DNA Polymerase (New England BioLabs, Catalog# M0491) with final concentration of 1X 5X Q5 Reaction Buffer, 1X 5X Q5 High GC Enhancer, 200μM dNTPs, 0.5μM forward and reverse primers, and 0.02 U/ul Q5 DNA Polymerase. After initial 45 seconds of denaturing at 98°C, 30 cycles at 2-step temperatures involving 15 seconds denaturing at 98°C and 8 minutes annealing + extension at 72°C were done to carry out the entire amplification. The final PCR product was stabilized at 72°C for 10 minutes and incubated at 4°C until analysis.

The entire ~9kb PCR fragments were sequenced using Pacific Biosciences SMRT Sequencing Technology and DNA Library was built by using SMRTbell Template Preparation Reagent Kits 1.0 with 3ug purified (Promega Wizard® SV Gel and PCR clean-up System) PCR product that contains 28 near full-length PCR reactions, which were derived from plasma or PBMC from all 5 patients. The library was examined by 2100 Bioanalyzer for purity and concentration. SMRT sequencing was carried out using the P4C2 chemistry with 1x120min acquisition mode on the PacBio RS according to standard protocol [52,53]. Data was analyzed using an in-house developed computational code. The final product yielded 19 (median 5) near full-length HIV-1 genomes.

Gene sequences for the above chronic donors have been submitted to GenBank, serial numbers pending. Acute sequences have either previously been reported [54], or have pending serial numbers.

### Assessing CD4-mediated killing using 7-aminoactinomycin D (7-AAD) staining

7-AAD assay was performed according to a modified protocol based on a prior study [55]. PBMCs from autologous or complete HLA-II mismatched HIV seropositive donors were used as target cells. CD4^+^ T cells were isolated from the PBMCs by CD8-depletion (Dynabeads CD8, Invitrogen) and activated with PHA (5μg/mL) in the presence of IL-2 (100IU/mL) for 2 days. Similar to prior studies [56], activated CD4 targets (1x10^5^ cells) were CFSE-labeled and were pulsed with the relevant HIV-1 NAE or AE peptide at 10uM for 1h before co-culturing with the appropriate NAE or AE-specific CD4^+^ T-cell line for 24 hrs at various E/T ratios (0:1, 0.5:1, 1:1, and 1.5:1). After incubation, the cells were surface stained with anti-CD3-Pac Blue (BD Biosciences) and anti-CD4-Alexa780 (eBioscience) before washing and staining with 0.25ug of 7-AAD (BD Biosciences) for 20 min at 4°C. Using flow cytometry, antigen-specific killing was determined by comparing the percentage of 7-AAD^+^ CD4 T cells in the presence of effector CD4 line relative to that in the absence of effector line (S5 Fig).

### Statistical analysis

Statistical tests were carried out using Fisher’s exact test, paired t-test, and nonparametric Mann-Whitney U. GraphPad Prism software (version 5.0) was used to perform these analyses. A p value of <0.05 was considered statistically significant.

## Supporting Information

S1 Fig Flow cytometry gating strategy for a representative HIV-1 controller and a non-controller. Flow cytometry plots depicting purity of CD8 depletion for a controller (top panel) and a non-controller (bottom panel) are shown.
(TIFF)

S2 Fig Novel CD4+ T cell epitopes are restricted by predicted HLA-II alleles. Shown is the HLA class II restriction of (A) DRB1*13-restricted Gag peptide (sequence: CKTILKALGPAATLEEMMTA) and (B) DRB1*03-restricted Nef peptide (sequence: MRRAEPAADGVGAVSRDLEK) as determined in an IFN- ELISpot assay using PBMC samples from chronically HIV infected patients–C1 (DRB1*13:02, DRB1*15:03, DQB1*03:03, DQB1*05:01) and NC1 (DRB1*03:01, DRB1*13:01, DQB1*02:01, DQB1*06:03). Effectors were CD4^+^ T cells expanded short term *in vitro* and the APC were transfected HLA-II expressing RM3 cells pulsed with the cognate peptide. The HLA-II matched and mismatched RM3 lines express DRB1*13:02 and DRB1*03:02, respectively, for restriction experiment done in (A), and DRB1*03:02 and DQB1*05:01, respectively, in (B). Error bars in (A) represent the SEM from duplicate experiments.
(TIFF)

S3 Fig Functional avidity of NAE and AE epitopes. Graphical representation of antigen sensitivity responses is shown in four NAE/AE pairs where the donor sequence matches the NAE, and for 2 NAE/AE pairs where the donor sequence matches the AE. Error bars represent the SEM from duplicate experiments. Wilcoxon matched-pairs signed rank test was used to determine statistical significance (*).
(TIFF)

S4 Fig Epitope-specific CD4 T cells produce cytolytic molecules. **A)** Representative flow cytometry plots on IFN-γ/CD107a and IFN-γ/Granzyme A producing CD4 T-cell responses to a DQB1*02-restricted non-adapted epitope (NAE) from a chronically infected patient are shown. **(B)** The overall *ex vivo* polyfunctionality of cytokine/effector molecule production of CD4 responses (5 functions) to 5 pairs of non-adapted (NAE) and adapted (AE) epitopes from 4 patients (2 controllers and 2 non-controllers) was evaluated using ICS and SPICE and PESTLE software. IFNg = IFN-γ; IL2 = IL-2; TNFa = TNF-α; CD107a = CD107a; Gran A = Granzyme A
(TIFF)

S5 Fig Representative 7-AAD killing assay for NAE and AE-specific CD4 T cells. Flow cytometry plots on 7-AAD staining of apoptotic target cells for a representative DQB1*06:11 restricted NAE/AE CD4 mediated killing in patient C6 are shown.
(TIFF)

S1 Table Clinical and demographic features of chronically HIV-1 infected cohort used in this study. (PDF)

S2 Table Clinical and demographic features of acutely HIV-1 infected cohort in the study. (PDF)

S3 Table HLA class II epitopes with the predicted polymorphisms that were evaluated for immunogenicity. (PDF)

S4 Table Aligning predicted non-adapted and adapted epitope sequences with autologous viral sequences in controllers (C) and non-controllers (NC). (PDF)

S5 Table Example of HLA-II relevant epitopes tested in acute patient (PHI-4). (PDF)

S6 Table Possible HLA-I linkage with HLA-II associated HIV-1 polymorphisms. (PDF)
