# Supplementary figures and images for: HLA Class-II Associated HIV Polymorphisms Predict Escape from CD4+ T Cell Responses
Source: PLoS Pathog. 2015 Aug 24;11(8):e1005111. doi: 10.1371/journal.ppat.1005111 (PMC4547780; doi:10.1371/journal.ppat.1005111)

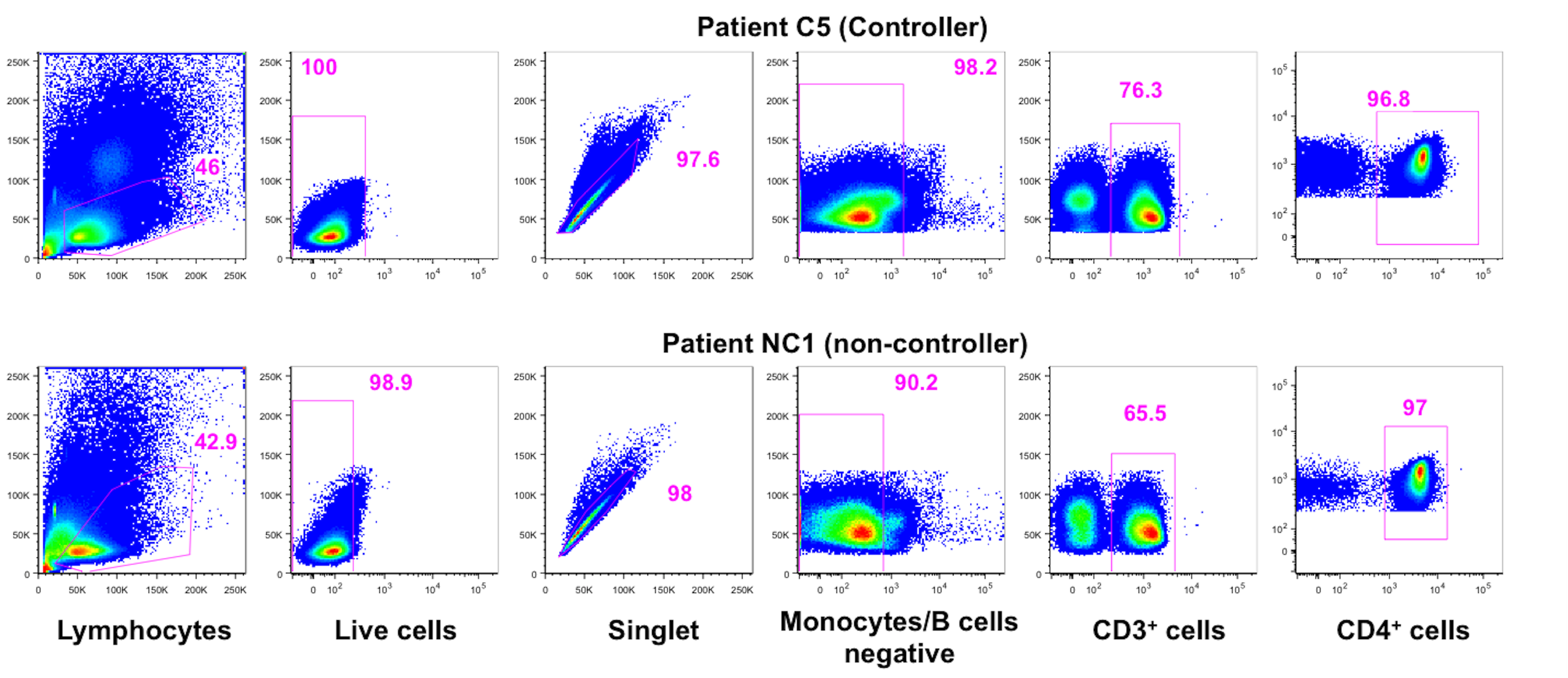

Supplement: S1 Fig — Flow cytometry plots depicting purity of CD8 depletion for a controller (top panel) and a non-controller (bottom panel) are shown. (TIFF) [file ppat.1005111.s001.tiff]

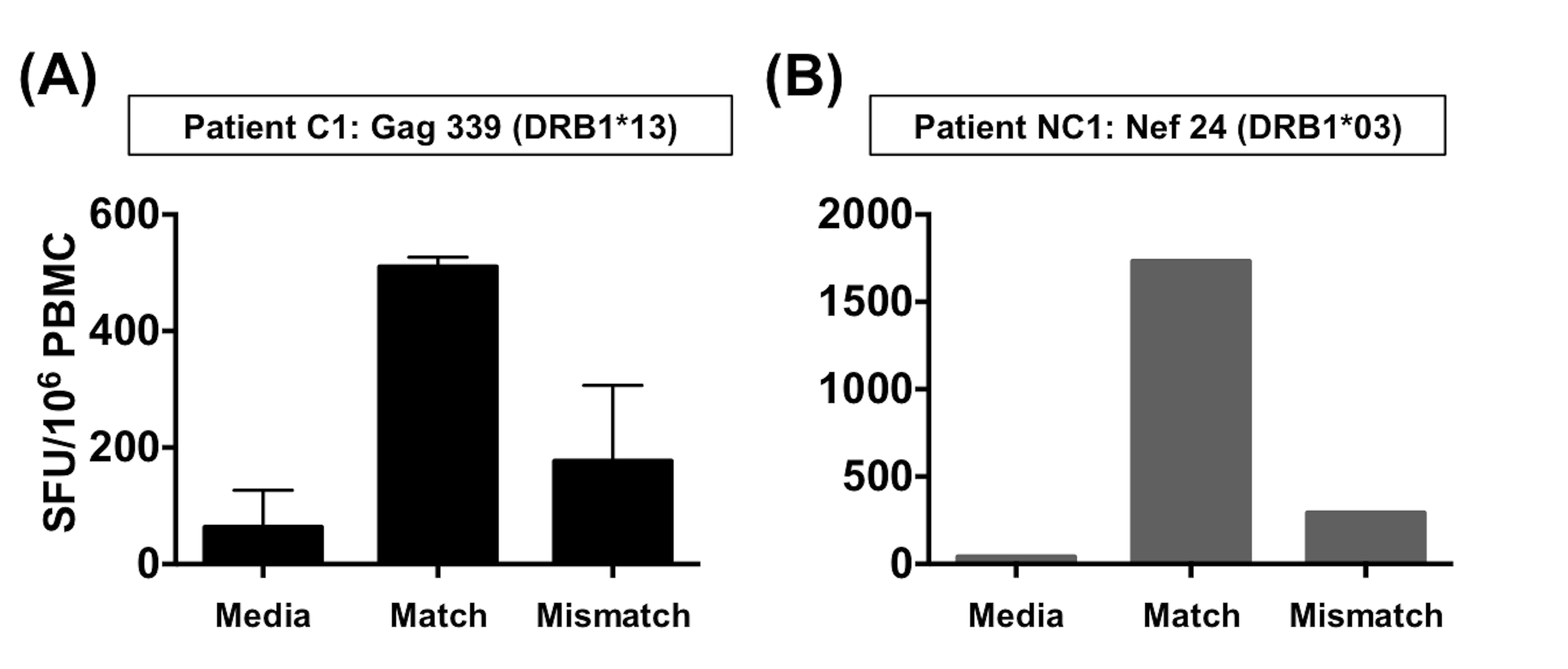

Supplement: S2 Fig — Shown is the HLA class II restriction of (A) DRB1*13-restricted Gag peptide (sequence: CKTILKALGPAATLEEMMTA) and (B) DRB1*03-restricted Nef peptide (sequence: MRRAEPAADGVGAVSRDLEK) as determined in an IFN- ELISpot assay using PBMC samples from chronically HIV infected patients–C1 (DRB1*13:02, DRB1*15:03, DQB1*03:03, DQB1*05:01) and NC1 (DRB1*03:01, DRB1*13:01, DQB1*02:01, DQB1*06:03). Effectors were CD4+ T cells expanded short term in vitro and the APC were transfected HLA-II expressing RM3 cells pulsed with the cognate peptide. The HLA-II matched and mismatched RM3 lines express DRB1*13:02 and DRB1*03:02, respectively, for restriction experiment done in (A), and DRB1*03:02 and DQB1*05:01, respectively, in (B). Error bars in (A) represent the SEM from duplicate experiments. (TIFF) [file ppat.1005111.s002.tiff]

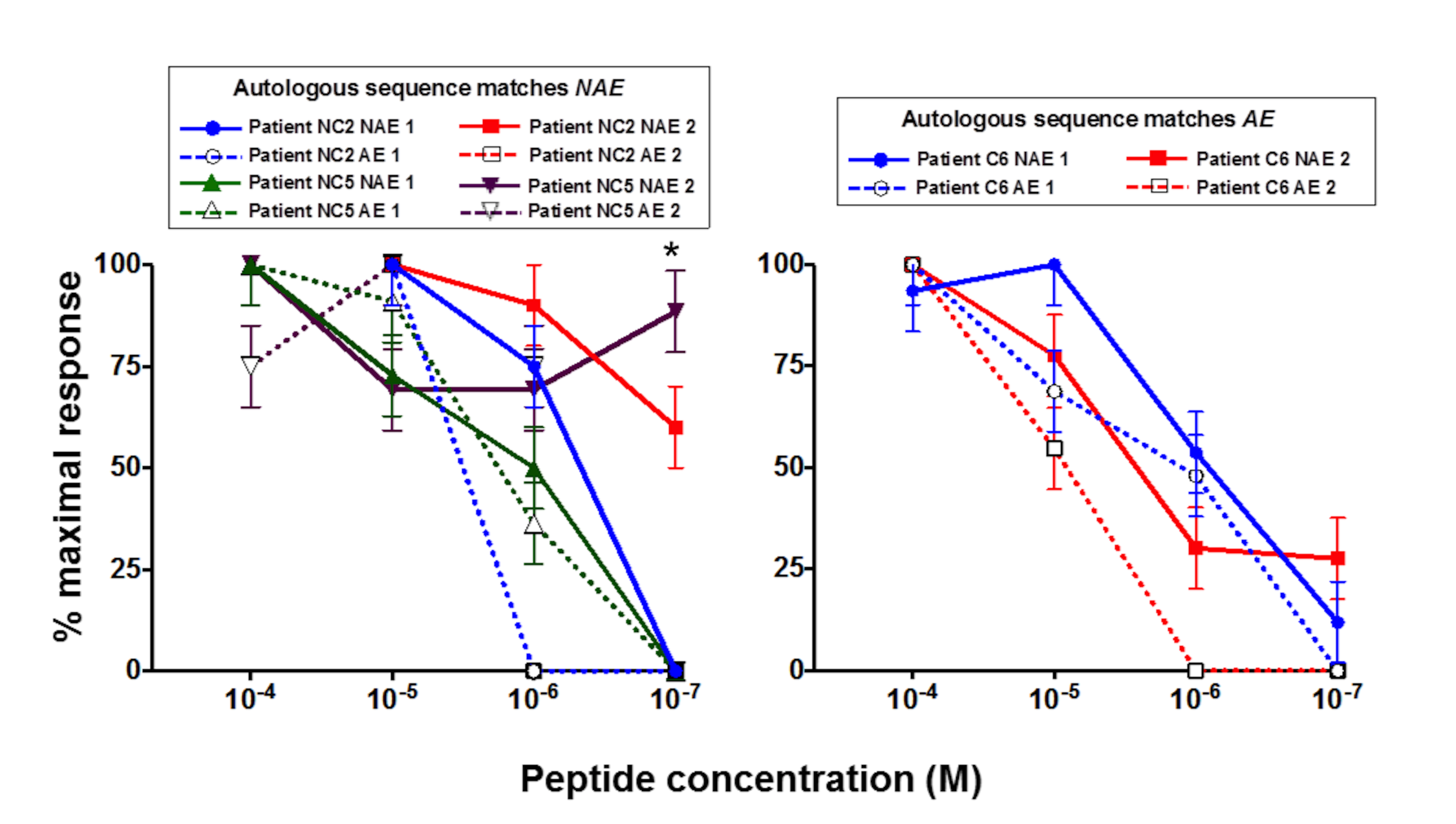

Supplement: S3 Fig — Graphical representation of antigen sensitivity responses is shown in four NAE/AE pairs where the donor sequence matches the NAE, and for 2 NAE/AE pairs where the donor sequence matches the AE. Error bars represent the SEM from duplicate experiments. Wilcoxon matched-pairs signed rank test was used to determine statistical significance (*). (TIFF) [file ppat.1005111.s003.tiff]

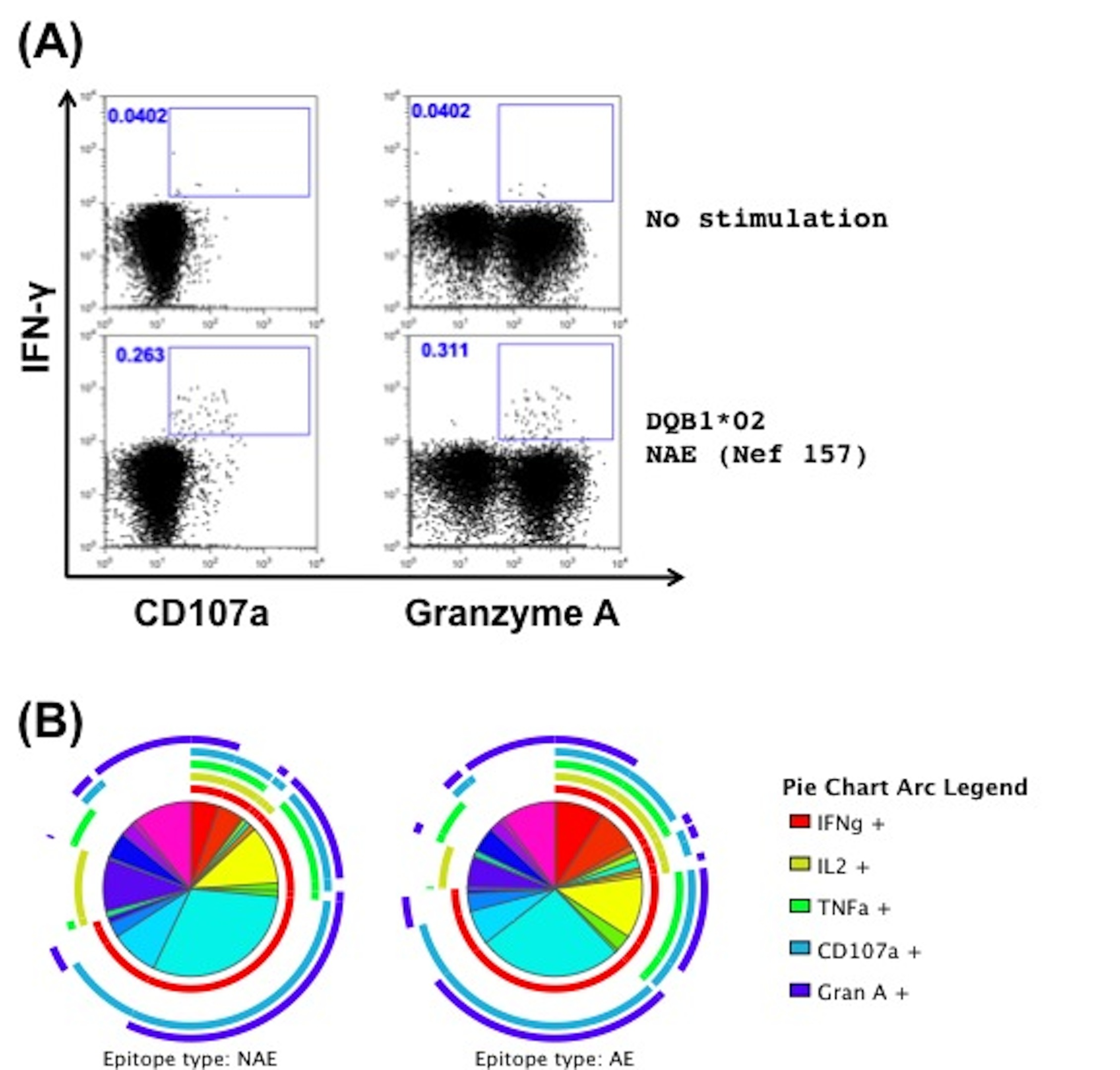

Supplement: S4 Fig — A) Representative flow cytometry plots on IFN-γ/CD107a and IFN-γ/Granzyme A producing CD4 T-cell responses to a DQB1*02-restricted non-adapted epitope (NAE) from a chronically infected patient are shown. (B) The overall ex vivo polyfunctionality of cytokine/effector molecule production of CD4 responses (5 functions) to 5 pairs of non-adapted (NAE) and adapted (AE) epitopes from 4 patients (2 controllers and 2 non-controllers) was evaluated using ICS and SPICE and PESTLE software. IFNg = IFN-γ; IL2 = IL-2; TNFa = TNF-α; CD107a = CD107a; Gran A = Granzyme A (TIFF) [file ppat.1005111.s004.tiff]

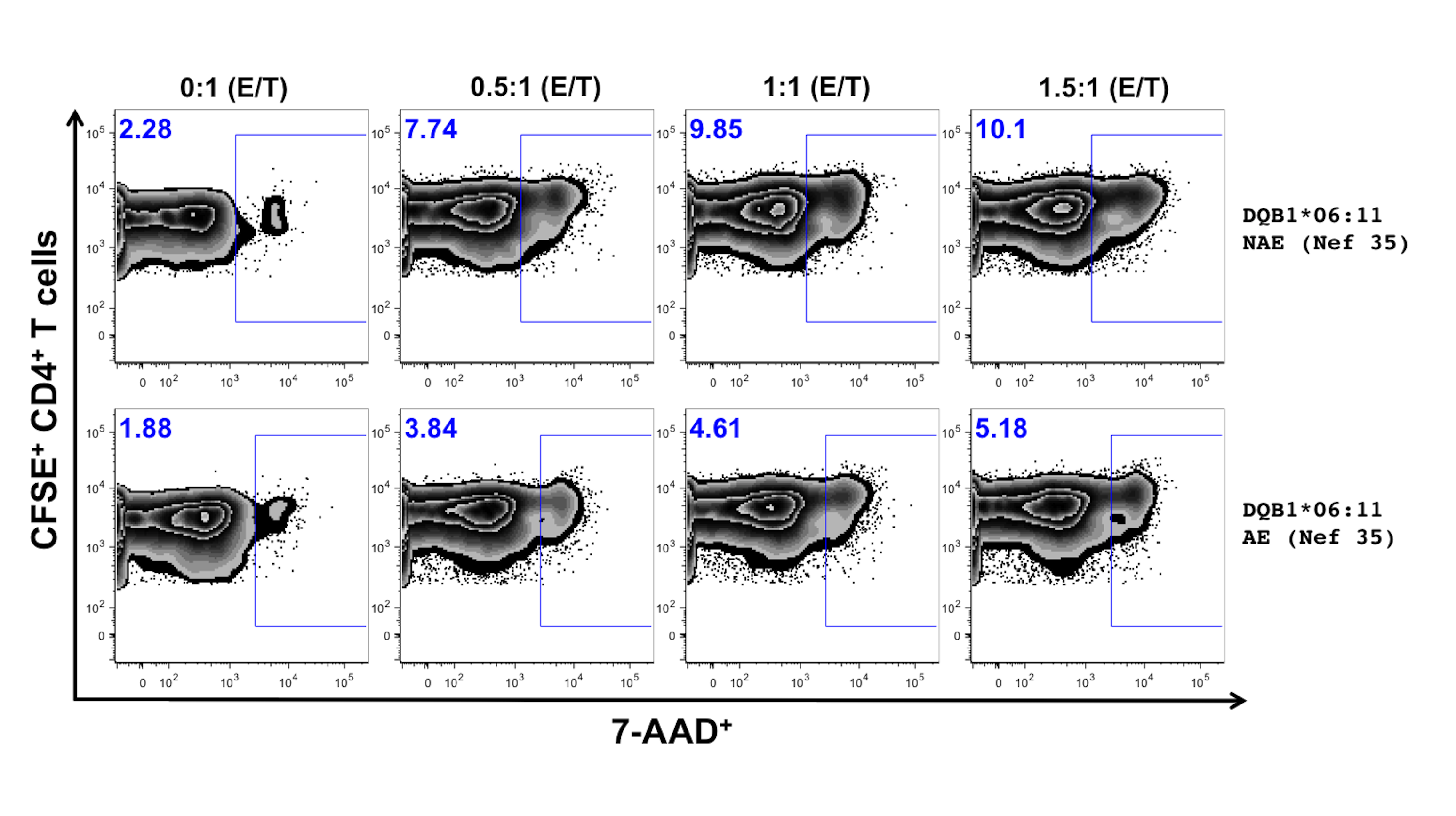

Supplement: S5 Fig — Flow cytometry plots on 7-AAD staining of apoptotic target cells for a representative DQB1*06:11 restricted NAE/AE CD4 mediated killing in patient C6 are shown. (TIFF) [file ppat.1005111.s005.tiff]
