# Supplementary material for: HLA Class-II Associated HIV Polymorphisms Predict Escape from CD4+ T Cell Responses
Source: PLoS Pathog. 2015 Aug 24;11(8):e1005111. doi: 10.1371/journal.ppat.1005111 (PMC4547780; doi:10.1371/journal.ppat.1005111)
Supplement: S1 Table — (PDF) [file ppat.1005111.s006.pdf]

Supplemental Table 1. Clinical and demographic features of chronically HIV-1 infected cohort used in this study

								HLA-II			
**Patient**	**Race[a]**	**Gender**	**Age**	**Risk factor[b]**	**Plasma VL[c]**	**CD4[d]**	**Status[e]**	**DRB1_AL1**	**DRB1_AL2**	**DQB1_AL1**	**DQB1_AL2**
**NC1**	CAU	M	52	MSM	24000	398	NC	03:01:01	13:01:01	02:01	06:03:01
**NC2**	CAU	M	54	MSM	23673	343	NC	01:01:01	14:01:01	05:01:01	05:03:01
**NC3**	AA	F	55	Heterosexual	5360	811	NC	03:02	03:02	04:02	04:02
**NC4**	AA	M	45	MSM	27000	621	NC	03:01:01	03:01:01	02:01	02:02
**NC5**	CAU	M	48	IVDU	38511	540	NC	01:01:01	15:01:01	05:01	06:02
**NC6**	CAU	M	50	MSM	18315	627	NC	04:01	13:02:01	03:02:01	06:03:01
**NC7**	AA	M	54	MSM	27231	122	NC	03:01:01	04:01:01	02:01	03:01:01
**NC8**	CAU	M	45	MSM	16121	531	NC	04:01:01	07:01	03:01:01	03:03:02
**NC9**	CAU	M	51	MSM	14920	848	NC	07:01:01	14:01:01	02:02	05:03:01
**NC10**	AA	M	37	MSM	15300	387	NC	03:01:01	08:04:01	02:01:01	03:01:01
**NC11**	AA	M	53	MSM	53400	284	NC	07:01	15:03:01	02:02	06:02:01
**NC12**	AA	M	54	MSM	44830	280	NC	01:01	15:01	05:01	06:02
**NC13**	AA	M	62	MSM	32657	717	NC	03:02	07:01	02:02	04:02
**NC14**	CAU	M	62	MSM	31837	275	NC	04:03:01	07:01:01	02:02	03:05:01
**C1**	AA	M	56	MSM	47	884	C	13:02:01	15:03:01	03:03:02	05:01:01
**C2**	AA	F	50	Heterosexual	47	1562	C	13:03:01	15:03:01	02:02	06:02:01
**C3**	AA	F	54	Heterosexual	280	470	C	13:01:01	13:01:01	05:01:01	06:08:01
**C4**	AA	M	44	MSM	329	1145	C	03:02:01	09:01:02	02:02	04:02
**C5**	AA	M	58	IVDU	47	777	C	13:02:01	13:03:01	02:02	06:09
**C6**	AA	M	48	Heterosexual	107	904	C	11:01:01	11:01:02	02:02	06:02:01
**C7**	CAU	M	37	IVDU	435	454	C	04:01:01	12:01:01	02:01:01	02:02
**C8**	CAU	M	49	MSM	561	848	C	04:03:01	14:01:01	03:02:01	05:03:01
**C9**	AA	M	48	Heterosexual	375	373	C	01:01	13:01	05:01:01	06:04:01
**C10**	AA	M	25	MSM	60	966	C	03:02:01	08:01:01	04:02	04:02
**C11**	AA	F	43	Heterosexual	1050	858	C	01:01:01	07:01:01	02:02	05:01:01
**C12**	CAU	M	55	MSM	21	837	C	01:01:01	04:07:01	03:01:01	05:01:01
**C13**	AA	M	58	MSM	83	698	C	13:01	15:03	06:02	06:03
**C14**	AA	F	49	Heterosexual	311	975	C	03:01:01	07:01:01	02:01:01	02:02

[a]CAU = Caucasian and AA = African-American; [b]MSM = Men who have sex with men and IVDU = IV drug user; [c]Plasma HIV-1 RNA copies/mL; [d]Absolute CD4 counts (cells/uL); [e]HIV controllers (C) are defined by < 2000 HIV-1 RNA copies/mL off ART and HIV non-controllers (NC) are defined by > 5000 HIV-1 RNA copies/mL off ART
