# Supplementary material for: HLA Class-II Associated HIV Polymorphisms Predict Escape from CD4+ T Cell Responses
Source: PLoS Pathog. 2015 Aug 24;11(8):e1005111. doi: 10.1371/journal.ppat.1005111 (PMC4547780; doi:10.1371/journal.ppat.1005111)
Supplement: S2 Table — (PDF) [file ppat.1005111.s007.pdf]

Supplemental Table 2. Clinical and demographic features of acutely HIV-1 infected cohort in the study

					HLA-II			
**Patient**	**Fiebig[a]**	**DPI[b]**	**Log VL[c]**	**CD4[d]**	**DRB1-AL1**	**DRB1_AL2**	**DQB1-AL1**	**DQB1-AL2**
**PHI-1**	II	31	5.53	415	07:01	15:01	02:02	06:02
**PHI-2**	II	31	4.34	479	01:03	15:01	05:01	06:02
**PHI-3**	III	51	5.40	395	04:04	07:01	02:02	03:02
**PHI-4**	I	42	4.74	932	03:02	13:01	04:02	06:04
**PHI-5**	I	33	7.26	230	01:02	15:03	02:02	05:01
**PHI-6**	II	32	4.68	671	15:03	15:03	02:02	06:02
**PHI-7**	I	23	7.00	237	15:01	15:01	06:02	06:02
**PHI-8**	I	30	5.23	286	04:01	04:04	03:01	03:02
**PHI-9**	I	16	6.45	719	04:05	12:01	03:02	05:01
**PHI-10**	II	21	6.00	472	13:01	15:01	06:02	06:09
**PHI-11**	I	20	6.68	164	11:04	11:04	05:02	05:02

[a]Fiebig stage during which SGA was done to define TFV; [b]DPI = days post infection (number of days between date of infection and sample collection); [c]Plasma HIV-1 RNA copies/mL; [d]Absolute CD4 counts (cells/uL)
