# Supplementary material for: HLA Class-II Associated HIV Polymorphisms Predict Escape from CD4+ T Cell Responses
Source: PLoS Pathog. 2015 Aug 24;11(8):e1005111. doi: 10.1371/journal.ppat.1005111 (PMC4547780; doi:10.1371/journal.ppat.1005111)
Supplement: S3 Table — (PDF) [file ppat.1005111.s008.pdf]

Supplemental Table 3. HLA class II epitopes with the predicted polymorphisms that were evaluated for immunogenicity

Protein	Position[a]	Amino acid	Potential HLA-II restriction	Epitope sequence[b]	Type[c]	Responder frequency[d]	Controller frequency[e]	Non-controller frequency[f]
Gag	112	K	DQB1*0603	QNKS**K**KKAQQAAADTGNSSQ	AE	4/28	3/14	1/14
				QNKS**K**KKAQQAAADTGNNSQ	AE	4/28	4/14	0/14
Gag	147	L	DQB1*02	YPIVQNLQGQMVHQAISPRT	NAE	4/28	3/14	1/14
				YPIVQNLQGQMVHQA**L**SPRT	AE	4/28	3/14	1/14
Gag	247	I	DQB1*06	RGSDIAGTTSTLQEQ**I**GWMT	AE	2/28	1/14	1/14
				RGSDIAGTTSTLQEQ**I**AWMT	AE	2/28	2/14	0/14
Gag	339	S	DRB1*13	CKTILKALGPAATLEEMMTA	NAE	5/28	4/14	1/14
				CKTILKALG**S**AATLEEMMTA	AE	3/28	2/14	1/14
Pol	17	H	DQB1*0201	FREDLAFPQGKAREFSSEQT	NAE	0/28	0/14	0/14
				FREDLAFPQGKAREF**H**SEQT	AE	2/28	2/14	0/14
Pol	68	T	DQB1*05	SFSFPQITLWQRPLVSIKIG	NAE	1/28	1/14	0/14
				SFSFPQITLWQRPLV**T**IKIG	AE	2/28	2/14	0/14
Pol	161	E	DQB1*0402	LTQIGCTLNFPISPIDTVPV	NAE	2/28	2/14	0/14
				LTQIGCTLNFPISPI**E**TVPV	AE	1/28	1/14	0/14
Pol	208	D	DQB1*05	EGKISKIGPENPYNTPVFAI	NAE	2/28	2/14	0/14
				EGKISKIGP**D**NPYNTPVFAI	AE	2/28	2/14	0/14
Pol	215	I	DRB1*08	YNTPVFAIKKKDSTKWRKLV	NAE	2/28	2/14	0/14
				YNTP**I**FAIKKKDSTKWRKLV	AE	2/28	2/14	0/14
Pol	333	V	DRB1*0102	KQNPDIVIYQYMDDLYVGSD	NAE	4/28	3/14	1/14
				KQNPD**V**VIYQYMDDLYVGSD	AE	2/28	2/14	0/14
Pol	362	K	DQB1*03	IEELRQHLLRWGFTTPDKKH	NAE	5/28	3/14	2/14
				IEELR**K**HLLRWGFTTPDKKH	AE	5/28	3/14	2/14
Pol	430	K	DQB1*0602	VGKLNWASQIYAGI**K**VKQLC	AE	1/28	1/14	0/14
				VGKLNWASQIYPGI**K**VKQLC	AE	1/28	1/14	0/14
Pol	433	Y	DQB1*0202	KLNWASQIYAGIKVKQLCKL	NAE	0/28	0/14	0/14
				KLNWASQIYAGIK**Y**KQLCKL	AE	1/28	1/14	0/14
Pol	490	D	DRB1*1001	DPSKDLIAEIQKQGQGQWTY	NAE	2/28	2/14	0/14
				DPSKDLIAEIQKQGQG**D**WTY	AE	2/28	2/14	0/14
Pol	499	D	DRB1*0901	TYQIYQEPFKNLKTGKYARM	NAE	2/28	2/14	0/14
				TYQIYQ**D**PFKNLKTGKYARM	AE	2/28	2/14	0/14
Pol	815	F	DQB1*0303	EAEVIPAETGQETAY**F**LLKL	AE	2/28	2/14	0/14
				EAEVIPAETGQETAY**F**ILKL	AE	1/28	1/14	0/14
				AETGQETAYYLLKLAGRWPV	NAE	3/28	3/14	0/14
				AETGQETAY**F**LLKLAGRWPV	AE	1/28	1/14	0/14
Pol	984	R	DQB1*0604	NSDIKVVPRRKAKIIKDYGK	NAE	2/28	2/14	0/14
				NSDIKVVPRRKAKII**R**DYGK	AE	2/28	2/14	0/14
				NSEIKVVPRRKAKII**R**DYGK	AE	2/28	2/14	0/14
Nef	16	I	DQB1*0201	MGGKWSKRSVVGWPTVRERM	NAE	3/28	3/14	0/14
				MGGKWSKRSVVGWPT**I**RERM	AE	2/28	2/14	0/14
Nef	20	L	DQB1*03	VRERMRRAEPAADGVGAVSR	NAE	3/28	3/14	0/14
				VRER**L**RRAEPAADGVGAVSR	AE	2/28	2/14	0/14
Nef	24	A	DRB1*03	MRRAEPAADGVGAVSRDLEK	NAE	3/28	2/14	1/14
				MRRA**A**PAADGVGAVSRDLEK	AE	2/28	2/14	0/14
Nef	35	R	DQB1*0611	GAVSQDLEKHGAITSSNTAA	NAE	3/28	3/14	0/14
				GAVS**R**DLEKHGAITSSNTAA	AE	3/28	3/14	0/14
				GAVA**R**DLEKHGAITSSNTAA	AE	3/28	3/14	0/14
Nef	45	N	DQB1*0201	EKHGAITSSNTAANNADCAW	NAE	4/28	3/14	1/14
				EKHGAIT**N**SNTAANNADCAW	AE	4/28	4/14	0/14
Nef	88	G	DQB1*04	VPLRPMTYKGALDLSHFLKE	NAE	4/28	2/14	2/14
				VPLRPMTYKGALDL**G**HFLKE	AE	2/28	2/14	0/14
Nef	104	Q	DQB1*05	GLIYSKKRQDILDLWVYHTQ	NAE	5/28	3/14	2/14
				GLIYS**Q**KRQDILDLWVYHTQ	AE	2/28	2/14	0/14
Nef	135	F	DQB1*0602	PGIRYPLTFGWCFKLVPVDP	NAE	4/28	3/14	1/14
				PGIR**F**PLTFGWCFKLVPVDP	AE	3/28	3/14	0/14
Nef	157	S	DRB1*1101	VEEANEGENNSLLHPMSLHG	NAE	3/28	2/14	1/14
				VEEA**S**EGENNSLLHPMSLHG	AE	2/28	1/14	1/14
				CFKLVPVEPEKVEEANEGEN	NAE	4/28	2/14	2/14
				CFKLVPVEPEKVEEA**T**EGEN	AE	4/28	2/14	2/14
Nef	168	L	DQB1*04	LLHPMSLHGMDDPEREVLVW	NAE	2/28	2/14	0/14
				LLHP**L**SLHGMDDPEREVLVW	AE	1/28	1/14	0/14
				EGENNSLLHPMSLHGMDDPE	NAE	1/28	1/14	0/14
				EGENNSLLHP**L**SLHGMDDPE	AE	1/28	1/14	0/14
Nef	188	G	DQB1*0602	VLVWKFDSRLAFHHMARELH	NAE	4/28	2/14	2/14
				VLVWKFDS**G**LAFHHMARELH	AE	3/28	2/14	1/14
Nef	192	R	DQB1*0201	KFDSRLAFHHMARELHPEYY	NAE	4/28	4/14	0/14
				KFDSRLAF**R**HMARELHPEYY	AE	3/28	3/14	0/14

[a] Amino acid (AA) position based on HXB2 numbering

[b] Amino acid (AA) polymorphism associated with the epitope's potential HLA-II restriction is bolded and underlined; all epitopes are novel in terms of AA sequence and HLA-II restriction

[c] AE = adapted epitope, NAE = non-adapted epitope

[d] Number of responders out of the total number of people tested for a particular peptide

[e] Number of responders out of the total number of controllers tested for a particular peptide

[f] Number of responders out of the total number of non-controllers tested for a particular peptide
