# Supplementary material for: HLA Class-II Associated HIV Polymorphisms Predict Escape from CD4+ T Cell Responses
Source: PLoS Pathog. 2015 Aug 24;11(8):e1005111. doi: 10.1371/journal.ppat.1005111 (PMC4547780; doi:10.1371/journal.ppat.1005111)
Supplement: S4 Table — (PDF) [file ppat.1005111.s009.pdf]

Supplemental Table 4. Aligning predicted non-adapted and adapted epitope sequences with autologous viral sequences in controllers (C) and non-controllers (NC)*

ID	Status	Protein	NAE	Mag/media	AE 1	Mag/media	AE 2	Mag/media
NC1	NC	Pol-RT	IEELRQHLLRWGFTTPDKKH<br>V----Q--------------	880/12.5	IEELRKHLLRWGFTTPDKKH<br>V----Q--------------	710/12.5		
		Pol-RT	KQNPDIVIYQYMDDLYVGSD<br>-----I--------------	10/12.5	KQNPDVVIYQYMDDLYVGSD<br>-----I--------------	5/12.5		
		Nef	GLIYSKKRQDILDLWVYHTQ<br>--V--QR--------I----	1045/12.5	GLIYSQKRQDILDLWVYHTQ<br>--V--QR--------I----	45/12.5		
		Nef	VEEANEGENNSLLHPMSLHG<br>----N-----------Q--	135/12.5	VEEASEGENNSLLHPMSLHG<br>----N-----------Q--	80/12.5		
		Nef	VLVWKFDSRLAFHHMARELH<br>----R---L--YR-------	930/12.5	VLVWKFDSGLAFHHMARELH<br>----R---L--YR-------	630/12.5		
		Nef	EKHGAITSSNTAANNADCAW<br>-------S-----H------	135/12.5	EKHGAITNSNTAANNADCAW<br>-------S-----H------	35/12.5		
		Nef	CFKLVPVEPEKVEEANEGEN<br>---------------N----	995/12.5	CFKLVPVEPEKVEEATEGEN<br>---------------N----	805/12.5		
		Nef	GAVSQDLEKHGAITSSNTAA<br>----R---------------	5/12.5	GAVSRDLEKHGAITSSNTAA<br>----R---------------	5/12.5	GAVARDLEKHGAITSSNTAA<br>---SR---------------	15/12.5
		Nef	VPLRPMTYKGALDLSHFLKE<br>-----------I--S-----	25/12.5	VPLRPMTYKGALDLGHFLKE<br>-----------I--S-----	5/12.5		
		Nef	KFDSRLAFHHMARELHPEYY<br>R---L--YR---------F	25/12.5	KFDSRLAFRHMARELHPEYY<br>R---L--YR---------F	25/12.5		

ID	Status	Protein	NAE	Mag/media	AE	Mag/media
NC2	NC	Gag p24	CKTILKALGPAATLEEMMTA<br>---------P----------	175/2.5	CKTILKALGSAATLEEMMTA<br>---------P----------	60/2.5
		Gag p24	YPIVQNLQGQMVHQAISPRT<br>------M--------I----	75/2.5	YPIVQNLQGQMVHQALSPRT<br>------M--------I----	60/2.5
		Pol-RT	KQNPDIVIYQYMDDLYVGSD<br>-----I--------------	95/2.5	KQNPDVVIYQYMDDLYVGSD<br>-----I--------------	5/2.5
		Pol-RT	IEELRQHLLRWGFTTPDKKH<br>-----Q--------------	15/2.5	IEELRKHLLRWGFTTPDKKH<br>-----Q--------------	0/2.5
		Nef	PGIRYPLTFGWCFKLVPVDP<br>--V-Y--C----------E-	75/2.5	PGIRFPLTFGWCFKLVPVDP<br>--V-Y--C----------E-	0/2.5
		Nef	VPLRPMTYKGALDLSHFLKE<br>--------------S-----	60/2.5	VPLRPMTYKGALDLGHFLKE<br>--------------S-----	10/2.5
		Nef	GLIYSKKRQDILDLWVYHTQ<br>-----Q----------H---	0/2.5	GLIYSQKRQDILDLWVYHTQ<br>-----Q----------H---	0/2.5
		Nef	LLHPMSLHGMDDPEREVLVW<br>----MN------------I-	15/2.5	LLHPLSLHGMDDPEREVLVW<br>----MN------------I-	0/2.5
		Nef	EGENNSLLHPMSLHGMDDPE<br>K----C----MN--------	0/2.5	EGENNSLLHPLSLHGMDDPE<br>K----C----MN--------	0/2.5

ID	Status	Protein	NAE	Mag/media	AE 1	Mag/media	AE 2	Mag/media
NC5	NC	Gag p24	CKTILKALGPAATLEEMMTA<br>---------P----------	15/5	CKTILKALGSAATLEEMMTA<br>---------P----------	10/5		
		Pol-RT	IEELRQHLLRWGFTTPDKKH<br>-----Q------L-------	180/5	IEELRKHLLRWGFTTPDKKH<br>-----Q------L-------	245/5		
		Pol-RT	KQNPDIVIYQYMDDLYVGSD<br>-----I--------------	30/5	KQNPDVVIYQYMDDLYVGSD<br>-----I--------------	15/5		
		Pol-Int	AETGQETAYYLLKLAGRWPV<br>---------F----------	15/5	AETGQETAYFLLKLAGRWPV<br>---------F----------	10/5		
		Nef	MRRAEPAADGVGAVSRDLEK<br>----E--------A-----R	105/5	MRRAAPAADGVGAVSRDLEK<br>----E--------A-----R	10/5		
		Nef	VPLRPMTYKGALDLSHFLKE<br>-----------V--S-----	85/5	VPLRPMTYKGALDLGHFLKE<br>-----------V--S-----	10/5		
		Nef	CFKLVPVEPEKVEEANEGEN<br>-----------I---NK---	210/5	CFKLVPVEPEKVEEATEGEN<br>-----------I---NK---	125/5		
		Nef	GLIYSKKRQDILDLWVYHTQ<br>-M-H-Q--------------	145/5	GLIYSQKRQDILDLWVYHTQ<br>-M-H-Q--------------	35/5		
		Nef	VLVWKFDSRLAFHHMARELH<br>--M-----H---R-------	220/5	VLVWKFDSGLAFHHMARELH<br>--M-----H---R-------	20/5		
		Nef	GAVSQDLEKHGAITSSNTAA<br>--A-R---R---------P-	5/5	GAVSRDLEKHGAITSSNTAA<br>--A-R---R---------P-	0/5	GAVARDLEKHGAITSSNTAA<br>--ASR---R---------P-	10/5
		Nef	EKHGAITSSNTAANNADCAW<br>-R-----S---P-T--A---	10/5	EKHGAITNSNTAANNADCAW<br>-R-----S---P-T--A---	20/5		
		Nef	PGIRYPLTFGWCFKLVPVDP<br>--V-Y-------------E-	25/5	PGIRFPLTFGWCFKLVPVDP<br>--V-Y-------------E-	10/5		
		Nef	VEEANEGENNSLLHPMSLHG<br>I---NK---HC------Q--	45/5	VEEASEGENNSLLHPMSLHG<br>I---NK---HC------Q--	0/5		
		Nef	KFDSRLAFHHMARELHPEYY<br>----H---R-----------	0/5	KFDSRLAFRHMARELHPEYY<br>----H---R-----------	20/5		

ID	Status	Protein	NAE	Mag/media	AE 1	Mag/media	AE 2	Mag/media
C4	C	Gag p24	CKTILKALGPAATLEEMMTA<br>---------P----------	210/7.5	CKTILKALGSAATLEEMMTA<br>---------P----------	25/7.5		
		Gag p24	YPIVQNLQGQMVHQAISPRT<br>--------------SL----	70/7.5	YPIVQNLQGQMVHQALSPRT<br>--------------SL----	90/7.5		
		Pol-RT	IEELRQHLLRWGFTTPDKKH<br>-----Q--------------	190/7.5	IEELRKHLLRWGFTTPDKKH<br>-----Q--------------	100/7.5		
		Pol-RT	KQNPDIVIYQYMDDLYVGSD<br>-----I--------------	20/7.5	KQNPDVVIYQYMDDLYVGSD<br>-----I--------------	5/7.5		
		Nef	GAVSQDLEKHGAITSSNTAA<br>----R---R---V-------	60/7.5	GAVSRDLEKHGAITSSNTAA<br>----R---R---V-------	40/7.5	GAVARDLEKHGAITSSNTAA<br>---SR---R---V-------	25/7.5
		Nef	GLIYSKKRQDILDLWVYHTQ<br>-----Q--------------	125/7.5	GLIYSQKRQDILDLWVYHTQ<br>-----Q--------------	15/7.5		
		Nef	VLVWKFDSRLAFHHMARELH<br>--E-R---R----V--D--	305/7.5	VLVWKFDSGLAFHHMARELH<br>--E-R---R----V--D--	170/7.5		
		Nef	MRRAEPAADGVGAVSRDLEK<br>---TQ-------------R	110/7.5	MRRAAPAADGVGAVSRDLEK<br>---TQ-------------R	30/7.5		
		Nef	EKHGAITSSNTAANNADCAW<br>-R---V-S-----T--A---	70/7.5	EKHGAITNSNTAANNADCAW<br>-R---V-S-----T--A---	55/7.5		
		Nef	CFKLVPVEPEKVEEANEGEN<br>--------KD-I---N-E--	265/7.5	CFKLVPVEPEKVEEATEGEN<br>--------KD-I---N-E--	90/7.5		
		Nef	KFDSRLAFHHMARELHPEYY<br>R-------H-V--D------	175/7.5	KFDSRLAFRHMARELHPEYY<br>R-------H-V--D------	50/7.5		
		Nef	VPLRPMTYKGALDLSHFLKE<br>---------A-V--S--I--	35/7.5	VPLRPMTYKGALDLGHFLKE<br>---------A-V--S--I--	15/7.5		

ID	Status	Protein	NAE	Mag/media	AE 1	Mag/media	AE 2	Mag/media
C6	C	Gag p24	CKTILKALGPAATLEEMMTA<br>---------P----------	390/75	CKTILKALGSAATLEEMMTA<br>---------P----------	325/75		
		Gag p24	YPIVQNLQGQMVHQAISPRT<br>------A--------I----	1000/75	YPIVQNLQGQMVHQALSPRT<br>------A--------I----	435/75		
		Gag p6	FREDLAFPQGKAREFSSEQT<br>---N------E----PPK-A	130/75	FREDLAFPQGKAREFHSEQT<br>---N------E----PPK-A	1080/75		
		Pol-PR	SFSFPQITLWQRPLVSIKIG<br>---------------T----	470/75	SFSFPQITLWQRPLVTIKIG<br>---------------T----	795/75		
		Pol-RT	EGKISKIGPENPYNTPVFAI<br>---------E------I---	380/75	EGKISKIGPDNPYNTPVFAI<br>---------E------I---	365/75		
		Pol-RT	YNTPVFAIKKKDSTKWRKLV<br>----I---------------	485/75	YNTPIFAIKKKDSTKWRKLV<br>----I---------------	355/75		
		Pol-RT	IEELRQHLLRWGFTTPDKKH<br>-----Q--------------	935/75	IEELRKHLLRWGFTTPDKKH<br>-----Q--------------	975/75		
		Pol-RT	KQNPDIVIYQYMDDLYVGSD<br>DK--EII-------------	465/75	KQNPDVVIYQYMDDLYVGSD<br>DK--EII-------------	540/75		
		Pol-RT	TYQIYQEPFKNLKTGKYARM<br>-F----E------------T	270/75	TYQIYQDPFKNLKTGKYARM<br>-F----E------------T	1270/75		
		Pol-RT	DPSKDLIAEIQKQGQGQWTY<br>----------------Q--F	1325/75	DPSKDLIAEIQKQGQGDWTY<br>----------------Q--F	1030/75		
		Pol-RT	LTQIGCTLNFPISPIDTVPV<br>---------------E----	530/75	LTQIGCTLNFPISPIETVPV<br>---------------E----	780/75		
		Pol-RT	KLNWASQIYAGIKVKQLCKL<br>---------P---V------	170/75	KLNWASQIYAGIKYKQLCKL<br>---------P---V------	205/75		
		Pol-Int	AETGQETAYYLLKLAGRWPV<br>---------F----------	315/75	AETGQETAYFLLKLAGRWPV<br>---------F----------	285/75		
		Pol-Int	NSDIKVVPRRKAKIIKDYGK<br>--E------------R----	830/75	NSDIKVVPRRKAKIIRDYGK<br>--E------------R----	895/75	NSEIKVVPRRKAKIIRDYGK<br>---------------R----	995/75
		Nef	VRERMRRAEPAADGVGAVSR<br>---KIKQT----E---PA--	1195/75	VRERLRRAEPAADGVGAVSR<br>---KIKQT----E---PA--	680/75		
		Nef	MRRAEPAADGVGAVSRDLEK<br>IKQTE---E---PA------	665/75	MRRAAPAADGVGAVSRDLEK<br>IKQTE---E---PA------	425/75		
		Nef	GLIYSKKRQDILDLWVYHTQ<br>--VW-Q---E------H---	810/75	GLIYSQKRQDILDLWVYHTQ<br>--VW-Q---E------H---	775/75		
		Nef	LLHPMSLHGMDDPEREVLVW<br>----M-Q---E---K-----	460/75	LLHPLSLHGMDDPEREVLVW<br>----M-Q---E---K-----	175/75		
		Nef	EGENNSLLHPMSLHGMDDPE<br>T----C----M-Q---E---	980/75	EGENNSLLHPLSLHGMDDPE<br>T----C----M-Q---E---	960/75		
		Nef	MGGKWSKRSVVGWPTVRERM<br>---------RD--SAV--KI	835/75	MGGKWSKRSVVGWPTIRERM<br>---------RD--SAV--KI	770/75		
		Nef	VEEANEGENNSLLHPMSLHG<br>--Q-NT----C------Q--	675/75	VEEASEGENNSLLHPMSLHG<br>--Q-NT----C------Q--	725/75		
		Nef	CFKLVPVEPEKVEEANEGEN<br>-------D-DV--Q-NT---	1395/75	CFKLVPVEPEKVEEATEGEN<br>-------D-DV--Q-NT---	1540/75		
		Nef	PGIRYPLTFGWCFKLVPVDP<br>----Y---------------	285/75	PGIRFPLTFGWCFKLVPVDP<br>----Y---------------	395/75		
		Nef	GAVSQDLEKHGAITSSNTAA<br>-PA-R---------T-----	465/75	GAVSRDLEKHGAITSSNTAA<br>-PA-R---------T-----	655/75	GAVARDLEKHGAITSSNTAA<br>-PASR---------T-----	640/75
		Nef	EKHGAITSSNTAANNADCAW<br>-------T--------N---	745/75	EKHGAITNSNTAANNADCAW<br>-------T--------N---	815/75		
		Nef	KFDSRLAFHHMARELHPEYY<br>--------H---------F-	1165/75	KFDSRLAFRHMARELHPEYY<br>--------H---------F-	1030/75		
		Nef	VLVWKFDSRLAFHHMARELH<br>--------R-----------	1485/75	VLVWKFDSGLAFHHMARELH<br>--------R-----------	1200/75		
		Nef	VPLRPMTYKGALDLSHFLKE<br>---------A-F--S-----	130/75	VPLRPMTYKGALDLGHFLKE<br>---------A-F--S-----	180/75		

*Amino acids at the HLA-II associated polymorphic sites are either highlighted in green (non-adapted) or in red (adapted); the corresponding autologous viral sequence is listed below each tested epitope sequence; epitopes matching the autologous viral sequences at the polymorphic sites are highlighted in gray; immunogenic CD4 T-cell responses assessed by IFN-γ ELISpot are underlined; if two AE variants are tested along with the NAE peptide, then one of the AE peptides is listed under "AE 1," and the other is listed under "AE 2."
