# Supplementary material for: HLA Class-II Associated HIV Polymorphisms Predict Escape from CD4+ T Cell Responses
Source: PLoS Pathog. 2015 Aug 24;11(8):e1005111. doi: 10.1371/journal.ppat.1005111 (PMC4547780; doi:10.1371/journal.ppat.1005111)
Supplement: S5 Table — (PDF) [file ppat.1005111.s010.pdf]

Supplemental Table 5. Example of HLA-II relevant epitopes tested in acute patient PHI-4

HLA-II: DRB1*03:02/13:01, DQB1*04:02/06:04			
Protein	Predicted HLA-II restriction	Epitope sequence[a]	Type[b]
Gag	DRB1*13	CKTILKALGPAATLEEMMTA	NAE
Nef	DQB1*04:02	LLHPMSLHGMDDPEREVLVW	NAE
Nef	DRB1*03	MRRAEPAADGVGAVSRDLEK	NAE
Nef	DQB1*06:02	VLVWKFDSRLAFHHMARELH	NAE
Pol	DQB1*04:02	LTQIGCTLNFPISPI**<u>E</u>**TVPV	AE
Gag	DQB1*06	RGSDIAGTTSTLQEQ**<u>I</u>**GWMT	AE
Nef	DQB1*06:02	PGIR**<u>F</u>**PLTFGWCFKLVPVDP	AE
Pol	DQB1*06:02	VGKLNWASQIYPGI**<u>K</u>**VKQLC	AE
Gag	DQB1*06:03	QNKS**<u>K</u>**KKAQQAAADTGNNSQ	AE
Pol	DQB1*06:04	NSDIKVVPRRKVKII**<u>R</u>**DYGK	AE
Nef	DQB1*06:11	GAVS**<u>R</u>**DLEKHGAITSSNTAA	AE

[a] Amino acid (AA) polymorphism associated with the epitope's potential HLA-II restriction is bolded and underlined; all epitopes are based on the HLA-II and TFV sequence of PHI-4

[b] AE = adapted epitope and NAE = non-adapted epitope
