# Supplementary material for: HLA Class-II Associated HIV Polymorphisms Predict Escape from CD4+ T Cell Responses
Source: PLoS Pathog. 2015 Aug 24;11(8):e1005111. doi: 10.1371/journal.ppat.1005111 (PMC4547780; doi:10.1371/journal.ppat.1005111)
Supplement: S6 Table — (PDF) [file ppat.1005111.s011.pdf]

Supplemental Table 6. Possible HLA-I linkage with HLA-II associated HIV-1 polymorphisms

Protein	Position[a]	HLA-II polymorphism	HLA-II[b]	Possible HLA-I linkage	p-value[c]
Gag	112	K	DQB1*06:03	None	N/A
Gag	147	L	DQB1*02	***B*57:03***	***0.005***
Gag	247	I	DQB1*06	**A*68**	**0.125**
Gag	339	S	DRB1*13	None	N/A
Pol	17	H	DQB1*02:01	None	N/A
Pol	68	T	DQB1*05	None	N/A
Pol	161	E	DQB1*04:02	None	N/A
Pol	208	D	DQB1*05	None	N/A
Pol	215	I	DRB1*08	None	N/A
Pol	333	V	DRB1*01:02	None	N/A
Pol	362	K	DQB1*03	None	N/A
Pol	430	K	DQB1*06:02	None	N/A
Pol	433	Y	DQB1*02:02	None	N/A
Pol	490	D	DRB1*10:01	None	N/A
Pol	499	D	DRB1*09:01	None	N/A
Pol	815	F	DQB1*03:03	None	N/A
Pol	984	R	DQB1*06:04	**B*42:01**	**0.138**
Nef	16	I	DQB1*02:01	None	N/A
Nef	20	L	DQB1*03	None	N/A
Nef	24	A	DRB1*03	None	N/A
Nef	35	R	DQB1*06:11	None	N/A
Nef	45	N	DQB1*02:01	None	N/A
Nef	88	G	DQB1*04	None	N/A
Nef	104	Q	DQB1*05	**B*08:01**	**0.872**
Nef	135	F	DQB1*06:02	None	N/A
Nef	157	S/T	DRB1*11:01/DQB1*02:01	None	N/A
Nef	168	L	DQB1*04:02	None	N/A
Nef	188	G	DQB1*06:02	None	N/A
Nef	192	R	DQB1*02:01	**A*7401**	**0.269**

[a] Amino-acid (AA) position based on HXB2 numbering

[b] Predicted HLA-II allele associated with this AA polymorphism

[c] Significance of HLA-I/II linkage disequilibrium association
